# Directed Evolution of a Model Primordial Enzyme Provides Insights into the Development of the Genetic Code

**DOI:** 10.1371/journal.pgen.1003187

**Published:** 2013-01-03

**Authors:** Manuel M. Müller, Jane R. Allison, Narupat Hongdilokkul, Laurent Gaillon, Peter Kast, Wilfred F. van Gunsteren, Philippe Marlière, Donald Hilvert

**Affiliations:** 1 Laboratory of Organic Chemistry, ETH Zurich, Zurich, Switzerland; 2 Laboratory of Physical Chemistry, ETH Zurich, Zurich, Switzerland; 3 Institut de Génomique, CEA, Evry, France; 4 Heurisko USA, Newark, Delaware, United States of America; University of Pennsylvania, United States of America

## Abstract

The contemporary proteinogenic repertoire contains 20 amino acids with diverse functional groups and side chain geometries. Primordial proteins, in contrast, were presumably constructed from a subset of these building blocks. Subsequent expansion of the proteinogenic alphabet would have enhanced their capabilities, fostering the metabolic prowess and organismal fitness of early living systems. While the addition of amino acids bearing innovative functional groups directly enhances the chemical repertoire of proteomes, the inclusion of chemically redundant monomers is difficult to rationalize. Here, we studied how a simplified chorismate mutase evolves upon expanding its amino acid alphabet from nine to potentially 20 letters. Continuous evolution provided an enhanced enzyme variant that has only two point mutations, both of which extend the alphabet and jointly improve protein stability by >4 kcal/mol and catalytic activity tenfold. The same, seemingly innocuous substitutions (Ile→Thr, Leu→Val) occurred in several independent evolutionary trajectories. The increase in fitness they confer indicates that building blocks with very similar side chain structures are highly beneficial for fine-tuning protein structure and function.

## Author Summary

Proteins are linear polymers of a set of typically 20 different amino acid building blocks. The amino acid sequence—encoded by a genetic template—directs the folding of newly synthesized proteins into compact 3D structures and dictates the function of the protein product. Monomers containing distinct physico-chemical properties and geometries allow the formation of highly sophisticated architectures, and diverse functional groups enable enzymes to catalyze a plethora of chemical transformations. Nevertheless, the biochemical rationale for the exact composition (and particularly the redundancy) of the proteinogenic amino acid alphabet, which contains multiple building blocks that are chemically similar, remains enigmatic. By subjecting a simplified enzyme—constructed from only nine different amino acids—to directed evolution, we were able to investigate the impact of amino acid diversity on protein function. The most prolific variant selected in the course of the experiments expanded its amino acid alphabet, albeit through two surprisingly subtle mutations (isoleucine to threonine and leucine to valine). The mutations improve both stability and catalytic activity of the enzyme, thereby demonstrating that the presence of structurally similar amino acids specified by the genetic code is highly beneficial for protein fitness.

## Introduction

Nature uses monodisperse, sequence-specific polymers to perform a plethora of functions essential to survival. While ancient life forms are believed to have harnessed RNA molecules to accelerate vital metabolic reactions [1]–[3], proteins assumed most catalytic functions in the course of natural evolution. Proteins are generally superior to RNA as catalysts, principally due to their flexible backbone and chemically diverse side chains which enable construction of finely tuned active sites [4], [5]. However, at the dawn of the protein world, it is unlikely that all of the current proteinogenic α-amino acids were employed. The genetic code more plausibly started from a subset of contemporary building blocks – extant in the primordial soup or produced by early life forms – and subsequently expanded to meet increasingly challenging metabolic needs [6]. Such a scenario has three corollaries:

- *Amino acids were present on the primitive earth*. Experimental simulations of amino acid synthesis under primordial conditions [7] and analysis of meteorites [8] suggest that proteinogenic amino acids were indeed present in reasonable concentrations before the origin of life.
- *Proteins constructed from a primitive amino acid alphabet conveyed a selective advantage to their producers*. Even today, functional proteins constructed from simplified alphabets exist. For example, the antifreeze protein from winter flounder, which adopts a 48-residue helix, is composed of only seven different residues [9]. Artificial proteins, including simplified binding domains [10], [11] and enzymes [12] further corroborate that significant biological activity can be achieved with low amino acid diversity. In the most extreme case to date, a simplified version of the dimeric chorismate mutase (CM) from *Methanocaldococcus jannaschii* (MjCM) [13] was constructed entirely from only nine different amino acids [14], albeit with considerable losses in activity and stability compared to the wild-type enzyme.
- *The acquisition of new building blocks resulted in improved protein fitness* [*2*]. Amino acids containing functional groups, such as cysteine and histidine, clearly extend the functional repertoire of proteins by providing unique chemical reactivity. However, many amino acids that are proposed to have been added late to the genetic code, including Ile, Phe, Met, Asn, and Gln [15], do not contain reactive groups that enable novel chemistries. The impact of these residues on protein fitness is less clear.

Here, we experimentally evaluate this conundrum by monitoring the evolution of a simplified enzyme — the CM constructed from a 9-amino acid alphabet [14] — while allowing it to freely acquire any of the remaining proteinogenic amino acids. The active site of this model primordial enzyme contains essentially the same functional groups as wild-type CM enzymes, but it exhibits lower stability and probably suffers from suboptimal alignment of these residues. This system is therefore ideally suited to investigate how expansion of the amino acid repertoire might enhance catalytic sophistication of enzymes beyond the contribution of novel reactivities, thus providing insight into the development of the genetic code.

## Results

### A simplified chorismate mutase as a model for a primordial enzyme

**Figure 1:**
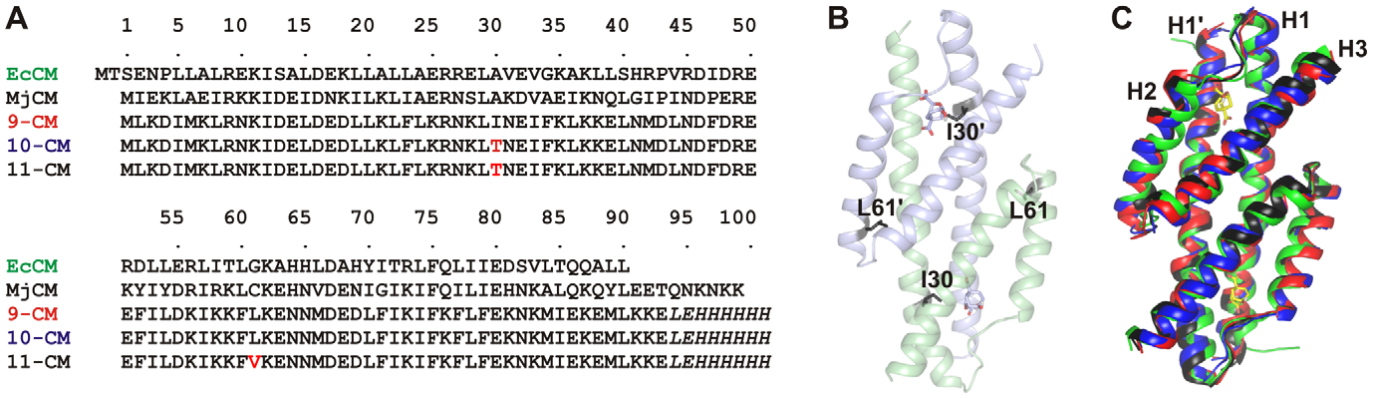
Comparisons of the structures of the CM variants. (A) Sequence alignment of natural and simplified mutases. EcCM refers to the CM domain of the bifunctional chorismate mutase-prephenate dehydratase enzyme from *E. coli*. Residue numbers given here correspond to MjCM positions for all CMs. (B) Ribbon representation of 9-CM with the locations of the residues that change identity in going from 9-CM to 11-CM shown as black sticks and the transition state analog (TSA) ligands shown as sticks colored according to atom type. (C) Homology model structures of the reduced amino-acid alphabet variants (9-CM: red, 10-CM: blue, 11-CM black) superimposed on the X-ray crystal structure of EcCM (green) [27] using Pymol. All structures were first energy-minimized in the GROMOS 53A6 force field.

MjCM is an all-helical, domain-swapped dimer that catalyzes the Claisen rearrangement of chorismate to prephenate, an essential transformation in the biosynthesis of aromatic amino acids [13]. The amino acid composition of a stable 93-residue variant [13] was previously simplified by two-stage combinatorial mutagenesis and *in vivo* selection. In the first step, the three helices in the enzyme were replaced with binary-patterned modules [16], [17] of randomized sequence, whereby only hydrophobic side chains were allowed for residues facing the interior of the protein and surface residues were constrained to polar building blocks [18]. Active enzymes in the degenerate libraries were then identified by their ability to complement the CM deficiency of a genetically engineered bacterial host [19]. One of the resulting 14-letter CMs was further simplified in a second step by systematically replacing loop residues and an active site glutamine with amino acids from the restricted alphabet [14]. *In vivo* selection finally yielded a variant, referred to here as 9-CM (Figure 1), composed of only nine different amino acids, namely the four polar (D, E, N, K) and four non-polar (F, I, L, M) residues from binary patterning, plus Arg, which is essential for binding the anionic substrate.

9-CM mimics many of the properties expected for a primitive enzyme. Despite dramatic simplification, it adopts a specific tertiary and quaternary fold as judged by CD spectroscopy and size-exclusion chromatography and catalyzes a metabolically important chemical transformation. The active site has all the functional groups needed for catalysis, including three arginines to bind the substrate carboxylates and other polar residues that contribute hydrogen-bonding interactions in the transition state. Nevertheless, 9-CM displays molten globular characteristics [14] and is 11 kcal/mol less stable and >1000-fold less active than the wild-type MjCM dimer. Because it was impossible to sample all sequences accessible in a nine amino acid alphabet when 9-CM was evolved, and due to the combinatorial fashion in which it was generated, the enzyme presumably resides at a *local* fitness maximum. These features make 9-CM an ideal starting point to explore how evolutionary expansion of the amino acid alphabet — with concomitant introduction of novel side chains — impacts protein structure and function.

Given the architecture of the genetic code, the evolvability of an enzyme is not solely based on its amino acid sequence. It also depends on codon usage, since different amino acid substitutions are accessible from different codons by point mutation [20]. Two different starting genes coding for 9-CM were therefore used for our evolution experiments: the gene obtained from the selection of 9-CM, enriched in NWV codons (where N = A,G,C,T; W = A,T; V = A,G,C) due to the mode of its construction [14], [18], and a synthetic version with a highly skewed nucleotide composition (89% A+T content). These are referred to as *n9-cm* and *AT9-cm*, respectively (Table S1). Besides offering alternative evolutionary trajectories, such distinctive starting points can potentially provide insight into the adaptation of genes.

### Evolution of the simplified mutase

To monitor the evolution of 9-CM, we first introduced the two genes encoding the simplified enzyme into the genome of a CM-deficient *E. coli* strain [19] under control of the highly regulable tetracycline promoter (P*_tet_*) [21] (see Materials and Methods for details). Not surprisingly, protein production is strongly reduced when genes for 9-CM are expressed chromosomally or from A+T-rich constructs, as compared to plasmid-based templates or more balanced nucleotide compositions (Figure S1, lanes 1, 4, and 5), causing a reduction in fitness. As a consequence, only the genomically reengineered strain harboring *n9-cm* grew under selective conditions in the presence of tetracycline, the activator of transcription. Chromosomal *AT9-cm* failed to complement the CM deficiency under all conditions tested.

To evolve the weakly complementing *n9-cm* strain, we resorted to automated cultivation in the GM3 device, an apparatus that enhances the growth rate of bacterial cells in suspension and counterselects the formation of biofilms [22]. The turbidostat regime, which maintains the optical density of a microbial population at a fixed setpoint by diluting the culture with pulses of fresh nutrient medium, is well suited for improving enzyme activity; cumulative fixation of adaptive mutations in the gene(s) encoding the enzyme(s) catalyzing the limiting metabolic reaction(s) is expected to occur over successive generations of the cultivated cell population. In addition to this growth control by infusion of fresh nutrient medium, transfer of the bacterial population is conducted cyclically between two cultivation chambers which alternatively undergo a transient period of sterilization with concentrated sodium hydroxide. In this way, any mutant cell that could adapt by attaching to any inner surface of the device and thus escape selection for faster growth in suspension is actively destroyed during every purging cycle of the device.

**Figure 2:**
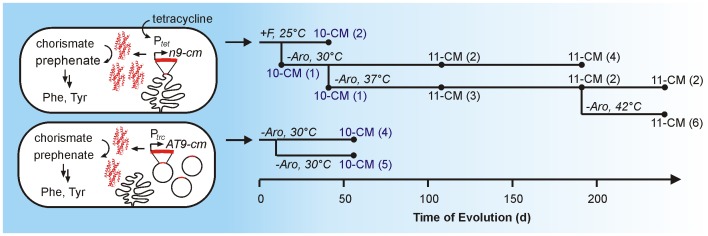
Schematic illustration of the CM evolution experiment. The cartoon on the left sketches out the key features of the bacterial hosts for the evolution of *n9-cm* (top) and *AT9-cm* (bottom). On the right, the time course of the evolution, including environmental conditions, are shown. Spheres denote time points where members of the population were sequenced. Among the few samples analyzed (number of sequences indicated in parentheses), the populations were homogeneous with respect to the CM gene. 10-CM and 11-CM contain the mutations I30T and I30T/L61V, respectively. The descriptor +F indicates that the full media composition (shown in Table S4) was used. Reduced CM medium also lacking Phe is denoted with –Aro (Table S4).

Figure 2 delineates, schematically, the history of the CM evolutionary process. In the early phase of the experiment, the growth medium was supplemented with high concentrations of tetracycline and phenylalanine but lacked tyrosine. These conditions permit growth of strains harboring weakly active CM variants. For later generations, L-Phe was omitted to increase selection stringency [19], while tetracycline was included at all stages of the experiment, as its omission would favor escape mutations in regulatory elements (i.e. the tet-regulon) over CM adaptation. At arbitrary intervals, the CM genes of several clones from the evolving population were sequenced. In the course of optimization, 9-CM first acquired the I30T mutation, yielding 10-CM, which subsequently acquired the L61V mutation to give 11-CM (Figure 1A, 1B, amino acid numbering according to MjCM). Each mutation occurred via a single base substitution at the DNA level, and extended the amino acid alphabet of the mutase by a single letter. The fact that these mutants arose spontaneously and outcompeted their respective parent suggests that both substitutions have beneficial effects on enzyme stability, *in vivo* concentration, activity, or some combination of these factors.

To enable analogous selection experiments with the AT-rich gene, *AT9-cm* was subcloned into a high-copy plasmid under the control of the strong *trc* promoter (pKECMT [23]). Increasing gene dosage was expected to compensate for possible gene expression problems, such as low rates of transcription/translation or low transcript stability. This construct indeed complemented the CM deficiency, and, after continuous evolution for 45 days in the turbidostat, faster growing variants were obtained. Isolation and sequencing of the responsible *AT9-cm* gene revealed a single AUU to ACU mutation, resulting in the same I30T substitution as observed for *n9-cm*. In addition, the helper plasmid pKIMP-UAUC had recombined with the plasmid coding for the CM variant at homologous promoter regions, as indicated by restriction digestions and DNA sequencing (Figure S7). Thus, two drastically different CM genes evolved by targeting the same locus, underscoring the importance of the I30T substitution.

**Figure 3:**
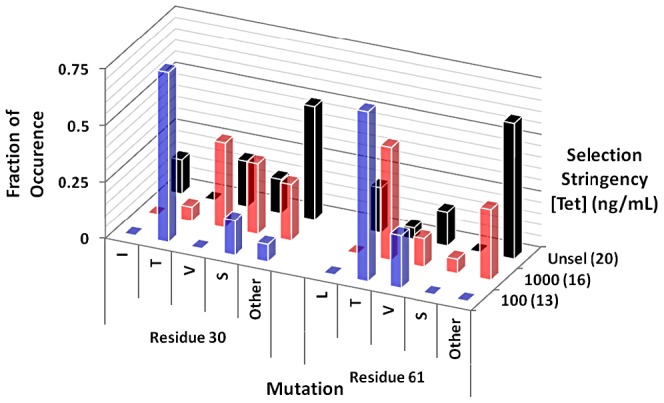
Overview of amino acid substitutions at mutation hot spots for cassette mutagenesis libraries. The fraction of mutations is plotted as a function of selection stringency (where a high tetracycline concentration [Tet] corresponds to low stringency; unselected library members are grown in the presence of L-Phe and L-Tyr) under which corresponding clones grew. Approximately 10% and 0.5% of all library members complemented the CM-deficiency at 1000 ng/mL and 100 ng/mL tetracycline, respectively. For the selected variants, the numbers of occurrences were normalized over the number of clones sequenced at the given selection stringency. Values in parentheses indicate the number of clones sequenced.

The observation that mutations occurred only at two very specific sites within the proteins suggests that there are surprisingly few hot-spot positions where substitutions can improve CM structure and function. Furthermore, the mutations of Ile30 to Thr and Leu61 to Val are surprisingly unassuming considering the other alphabet-expanding mutations that were accessible. To test whether this finding is an artifact of the genetic code's architecture, random cassette mutagenesis was performed at positions 30 and 61 simultaneously. A gene library constructed by PCR using degenerate oligonucleotides and *n9-cm* as a template was ligated into a high-copy plasmid (pKT [21]), and subjected to selection in CM-deficient cells. A comparison of the sequences of complementing clones under different selection stringencies with library members grown under non-selective conditions revealed pronounced residue preferences at the hotspot positions (Figure 3 and Figure S2, Tables S2 and S3). Under low selection stringency (1000 ng/mL tetracycline, Figure S2B), a range of small residues including Ser, Val, and Thr was selected. Upon increasing the selection stringency (100 ng/mL tetracycline, Figure S2C), a dramatic enrichment of Thr at each hot-spot position and, to a lesser extent, Ser (pos. 30) and Val (pos. 61) occurred in functional mutases. In contrast to the mutations Ile30Thr and Leu61Val, which are accessible via single-base substitution and which accumulated in the turbidostat selections, Leu61Thr requires two base substitutions, reducing its likelihood of spontaneous occurrence. In summary, regardless of starting gene and mode of mutagenesis, the simplified CM evolves in a defined way by conservative expansion of its building block repertoire dominated by subtle changes of residue size and polarity.

The evolutionary trajectory of *n9-cm* was scrutinized by characterizing 9-CM, 10-CM, and 11-CM *in vivo* and *in vitro*. To separate the effects of CM evolution from alternative mechanisms of strain adaptation to the selection pressure, we cloned the CM genes into high-copy plasmids (pKT) under the control of P*_tet_*. Four arbitrarily chosen CM variants that were subcloned after selection from the random cassette mutagenesis library were included for comparison. *E. coli* strains harboring these constructs were evaluated by *in vivo* complementation. The selection stringency of the assays was adjusted by varying the tetracycline concentration (Figure S3). While 9-CM only grows at concentrations of tetracycline >1000 ng/mL, the evolved variants still thrive when transcription is more strongly repressed (Figure S3A). Interestingly, none of the four CM variants selected from the random cassette mutagenesis library outperforms 11-CM in these complementation assays (Figure S3B). However, a positive control carrying a wild-type CM domain from *E. coli* (EcCM) [24] grows even in the absence of tetracycline.

Western blot analysis of *E. coli* cells producing 9-CM and its evolved variants revealed that expansion of the amino acid alphabet leads to increased CM production (Figure S1, lanes 1–3). Since the mutated codons found for 11-CM do not substantially differ from those of 9-CM with respect to codon usage, this result suggests that the evolved CMs fold into compact dimers more readily and are thus more resilient towards degradation *in vivo*.

### Biophysical characterization of evolved variants

Biophysical characterization of the original clone (9-CM) and the evolved variants (10-CM and 11-CM) yielded insight into the benefit provided by the two mutations. The proteins, equipped with a C-terminal His_6_ tag, were produced in a CM-deficient production strain [25] and purified by Ni-NTA affinity and size-exclusion chromatography. All variants eluted as dimers from the size-exclusion column, with a small fraction of aggregates in the more readily produced mutant proteins (Figure S4A).

**Figure 4:**
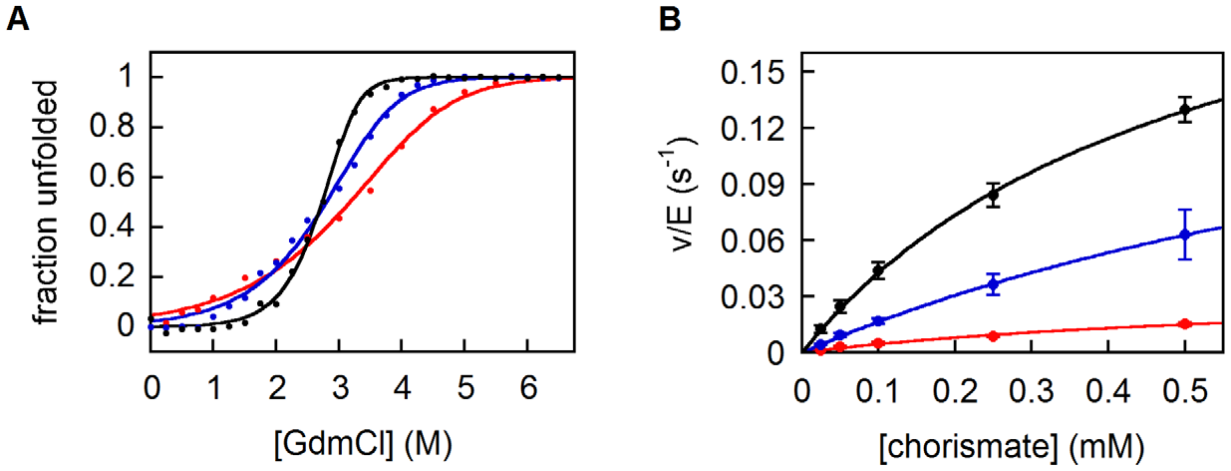
Biophysical characterization of CM variants. (A) Protein stabilities determined by chemical denaturation with GdmCl. Particularly for 9-CM (red) and 10-CM (blue), deviations from an ideal two-state unfolding, which are not unusual among helical bundle CMs [13], can be observed. To enable better comparison to 11-CM (black), the unfolding curves of all variants were nevertheless fit to a two-state model. (B) Michaelis-Menten kinetics of CM variants as in (A). The error bars represent standard deviations from 2 (10-CM, 11-CM) and 3 (9-CM) independent measurements.

Circular dichroism (CD) spectroscopy confirmed that all variants are highly helical (Figure S4B). CD was also used to assess stability (Figure S4C). While the parent 9-CM unfolds non-cooperatively upon heating, the evolved 10-CM and 11-CM show modest cooperativity, with midpoints of transition at 60 and 65°C, respectively. The free energies of unfolding Δ*G*_U_^0^(H_2_O), estimated from titrations with GdmCl, show the same trend (Figure 4A, Table 1). While the double mutant 11-CM is stabilized compared to the simpler variant 9-CM by more than 4 kcal/mol, it is still far less stable than natural CMs [13]. Limited proteolysis with trypsin was used to assess the compactness of the CM structures (Figure S5). 9-CM is cleaved readily over a timescale of minutes, whereas the evolved mutases show greater resilience towards proteolysis. For 11-CM, an intermediate corresponding to CM(1–92), i.e. a loss of the C-terminal residue plus the His_6_-tag (Figure S5B), is not degraded further by trypsin over the course of approximately 1 hour. These results parallel the equilibrium unfolding experiments, and highlight the fact that the I30T and the L61V mutations incrementally increase protein compactness.

**Table 1:** Comparison of natural and simplified chorismate mutases.

Protein	Δ*G*_U_^0^(H_2_O)	*m* d	*k*_cat_	*K*_m_	*k*_cat_ */K*_m_
	(kcal/mol)	(kcal/mol M^−1^)	(s^−1^)	(µM)	(M^−1^ s^−1^)
9-CM^a^	10.4±0.2	−1.01±0.05	0.035±0.005	700±160	49±3
10-CM	11.3±0.2	−1.45±0.05	0.21±0.11	1,200±800	170±7
11-CM	14.8±0.3	−2.78±0.08	0.26±0.02	510±80	500±80
EcCM^b^	20.5±0.4		69	300	230,000
MjCM^c^	26.9±0.9		5.7	41	140,000

a Values for 9-CM determined in this work. Activities were measured at 30°C in phosphate buffer, pH 6.5. At 20°C and pH 5, the activity is 3-fold higher, as expected for a mutase with a carboxylate at position 86 [42], [43], but still far lower than the activities determined in [14]. The values for *k*_cat_/*K*_m_ were found to be similar for two independently produced batches.

b Kinetic data (30°C, pH 7.5) are taken from [44], stabilities from [13].

c All values (30°C) are taken from [13].

d *m* is the slope of a plot of [GdmCl] vs Δ*G*_U_^0^, and relates to the degree of cooperativity of folding.

Expansion of the 9-amino acid alphabet positively affected catalytic properties as well (Figure 4B, Table 1). The steady-state parameters show that 10-CM and 11-CM are roughly 3-fold and 10-fold more active than 9-CM, respectively, mostly due to an increase in *k*_cat_. Nevertheless, wild-type mutases are again more sophisticated; their activities are several orders of magnitude higher. Although the two mutations improve both stability and activity of the protein, additional refinement will clearly be required to reach wild-type fitness levels.

### Molecular dynamics simulations

To rationalize the improvement in stability and activity achieved during the evolutionary trajectory, dimeric models of each variant were constructed and compared to EcCM (Figure 1C). A prototype 9-CM structure was created by homology modeling using I-TASSER [26] with the EcCM structure [27] as a reference. The structures for 10-CM and 11-CM were derived from that of 9-CM by *in silico* mutagenesis. One transition state analog (TSA) was placed into each active site. The structures were first energy-minimized and then subjected to a 10 ns molecular dynamics (MD) simulation at 293 K.

Despite having only ca. 25% sequence identity, the energy-minimized homology-modeled structures of the simplified CMs are all essentially identical to that of EcCM (Figure 1C). The structural stability, assessed in terms of secondary structure content and atom-positional root-mean-square deviation (rmsd) from the energy-minimized starting structure, is similar for EcCM and all of the evolved variants within the time frame of the simulations (Figure S6). The secondary structure is particularly well maintained, and comprises predominantly α-helical structure, in keeping with the results of the CD spectroscopy. Overall, these results show that the assumption of a similar structure for the reduced alphabet variants as for the wild-type EcCM is reasonable.

**Figure 5:**
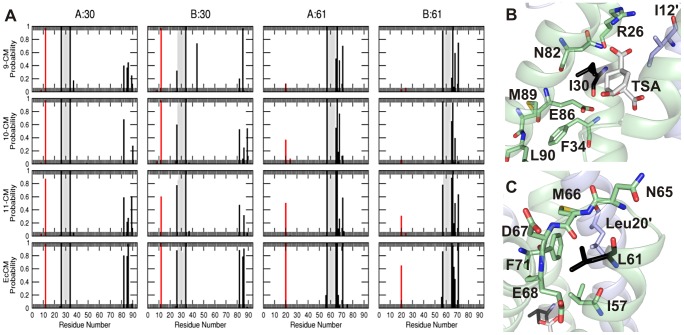
Local environment of hot-spot residues. (A) Probability that any atom of a residue comes within 0.6 nm of the Cα atom of residue 30 or 61 of subunit A or B during the 10 ns MD simulation of each CM variant as labeled. The probability for residues that lie within ±3 residues of residue 30 or 61 are shown in grey, that for residues further away but in the same subunit are in black, and that for residues in the alternate subunit are in red. (B) and (C) depict the local environment of residues 30 and 61 (black), respectively, in the energy-minimized structure of 9-CM.

While the global structure seems largely unperturbed in all of the simplified CMs, the MD simulations revealed how local interactions that are perturbed in the simplified CMs were restored during directed evolution. The environments of the residues that changed during the evolutionary trajectory were probed by calculating the fraction of the simulation frames in which at least one atom from a given residue falls within 0.6 nm of the Cα atom of residue 30 or 61 (Figure 5A). The primary interaction partners for residue 30 are residues 82–89 of the same subunit, and residue 12 of the alternate subunit (Figure 5B). The latter interaction occurs in nearly every structure saved during all simulations, whereas the interactions with residues 82–89 persist only for EcCM. Residue 61 of the wild-type EcCM interacts with residues 57 (helix 2), 65–68 (the loop connecting helices 2 and 3) and 71 (helix 3) of the same subunit, as well as Leu20 situated in helix 1 of the alternate subunit (Figure 1C and Figure 5C). In 9-CM, the local environments of residues 30 and 61 are partially rearranged. The probabilities of contact of Ile30 with Asn82 and Glu86 are diminished, in part at the expense of aberrant proximity to residues 89 and 90, or almost completely absent. Such distortions of the active site region may explain the reduced activity of the simplified mutases. In addition, the conformation of the loop between helices 2 and 3 appears to differ between EcCM and 9-CM, with helices 2 and 1′ being pushed apart in the latter (Figure 1C).

Upon mutation of Ile30 to Thr, helix 3 is repositioned, evident in the movement of residues 82 and 85 with respect to residue 30 and the loss of the interaction between Leu61 and Phe71 in 10-CM (Figure 5A). Although the introduction of a threonine potentially creates new possibilities for hydrogen bonding, the overall size of this residue is likely to have been more decisive given that natural AroQ class CMs typically have a Gly, Ala, or Val residue at this position [13]. Moreover, hydrogen-bonding interactions between Thr30 and the TSA were detected to a significant extent only in one subunit of 10-CM, but not in the other subunit of 10-CM or either subunit of 11-CM. The subsequent shortening of the side-chain of Leu61 to Val in 11-CM shifts helix 2, promoting contacts of residue 61 with helices 3 (Phe71) and 1′ (Leu20′) to better resemble the helix packing in EcCM. Interestingly, subunit symmetry appears to be correlated to CM activity, with the two subunits of more proficient CM variants being more similar than the subunits of their less evolved counterparts. In summary, the mutations I30T and L61V iteratively restore some of the structural features of highly active CMs, exemplified here by EcCM, lost upon simplification to 9-CM, bringing with them improvements in both the stability and the activity of the simplified CM.

## Discussion

In 1939, Ernest Vincent Wright wrote the novel “Gadsby”, a story of 50,000 words, without a single use of the letter “e”. In this so-called lipogram, Wright was able to tell a complete tale fluently, an achievement previously claimed to be impossible because of the abundance of “e”s in the English language. Still, without the past tense, definite articles, many numbers and pronouns, the text lacks some of the finesse that can be achieved with the entire alphabet.

The same principle holds true for biopolymers. Most simply, increasing the number of different building blocks in lattice models not only promotes uniqueness of folded structures, but also ameliorates folding kinetics [28], [29]. Regardless, functional polymers can be constructed with extremely small monomer diversity. For example, Joyce and co-workers evolved a ribozyme that lacks cytidine [30], and later extended their simplification to a two-nucleotide alphabet, the simplest possible [31]. To investigate the effect of added complexity, they evolved the cytidine-free ligase, allowing incorporation of the missing nucleotide [32]. With twelve changes (seven of which were mutations to C), the catalytic activity was improved 20-fold, predominantly by stabilizing the active conformation and fine-tuning the structure.

Here, we report how simplified proteins evolve when we let them access new building blocks. Upon prolonged *in vivo* selection for improved CM activity, a simplified CM constructed from a nine amino acid alphabet [14] acquired two mutations, I30T and L61V. These changes extend the alphabet to 11 letters, and improve the enzyme's stability by ca. 4 kcal/mol and its activity by a factor of 10. According to molecular dynamics simulations, the overall protein structure and the architecture of the active site are similar in all CM variants, but helix packing is distorted in the simplified enzyme 9-CM. The I30T and L61V mutations restore proper helix packing, which simultaneously increases protein stability and fine-tunes active site interactions and thus improves catalytic activity.

The evolutionary pathway for 9-CM is similar in three separate experiments starting from two structurally very different but synonymous genes, and two complementary strategies to introduce diversity, namely *in vivo* mutagenesis and random cassette mutagenesis. A transition resulting in the I30T mutation (ATC to ACC in *n9-cm*, ATT to ACT in *AT9-cm*) was rapidly selected in all evolution experiments conducted under competitive conditions. Subsequently, the mutation L61V was fixed in the long-term evolution of *n9-CM*. This outcome is paralleled when both positions are simultaneously varied using random cassette mutagenesis, whereby an enrichment of Thr and Ser at position 30, and Thr and Val at position 61 was observed in more active CMs. The rapid fixation of I30T from two distinct genetic templates indicates that the evolutionary pathway of the simplified enzyme is governed by a specific hotspot (residue 30) proximal to the active site of the protein. In addition, the mutations that arose are strikingly modest given that more drastic changes in physico-chemical properties were accessible at the hotspot positions, as well as throughout the entire protein. Random cassette mutagenesis confirmed, however, that these unspectacular mutations represented strongly competitive changes at the mutagenesis hotspots even in the absence of constraints due to the genetic code's architecture.

While the amino acid set of 9-CM differs from likely primordial alphabets [15], the conclusions drawn from this study are nevertheless relevant to the evolution of primitive functional proteins. The fact that all the mutations found in the CM selections extend the amino acid alphabet corroborates that 9-CM is situated at a local fitness maximum, thereby meeting the expectations for a primordial protein adapted to its producer's genetic code. Nevertheless, the mutations found in 11-CM do not contribute novel functionality to the active site. Instead, relatively modest changes in the building blocks, in this case shortening of the side chain by one methylene group (Leu to Val, Ile to Thr) and increased polarity as well as the potential to engage in H-bonding (Ile to Thr), fine-tune the enzyme's structure, and consequently its activity. Similar improvements presumably occurred simultaneously for many different primordial enzymes, greatly enhancing the fitness of early life forms.

Evolutionary experiments with proteins and organisms containing alternative or even expanded amino acid repertoires [33]–[36] suggest that the current genetic code is not an evolutionary dead end, but amenable to further natural and synthetic innovations. Our results show that incremental addition of even seemingly redundant building blocks can profoundly affect protein structure and activity, significantly enlarging the functional space of these biomolecules.

## Methods

A detailed description of experimental procedures is given in Text S1 of the supporting information.

### Methods summary

*In vivo* complementation assays for CM activity in KA12/pKIMP-UAUC [19], [21] as well as protein production from the CM-deficient *E. coli* strain KA13 and *in vitro* characterization were carried out as previously described [14]. Growth of strains harboring CM variants was assessed from single colony streak-outs using a phenotypic growth scale according to colony size (Table S5).

### Directed evolution in a pulse-feed alternating turbidostat

Genes encoding 9-CM under control of P*_tet_* were introduced into the genome of the CM-deficient *E. coli* strain KA12 at the *kdgK* locus (Figure S8) in a RecA-dependent manner according to published procedures [37]. *E. coli* cells harboring the chromosomally encoded *n9-cm* and the helper plasmid pKIMP-UAUC were grown in rich medium to an OD_600_ of 0.5, washed and resuspended in minimal medium and directly used to inoculate a 19.5-mL pulse-feed alternating turbidostat [22]. At regular intervals, a conditional pulse of fresh medium was delivered (if OD_600_>0.3), effecting a 10% dilution. Steady-state bacterial populations are thus expected to contain approximately 4–5×10^9^ cells. Once every 24 h, the growing culture was transferred to a backup chamber while the growth chamber was sterilized with NaOH, rinsed, and emptied.

Selections with the AT-rich gene were conducted in pKECMT [23] under control of the strong *trc* promoter. KA12/pKIMP-UAUC cells were transformed with pKECMT-AT-9 cm, adapted to selective CM-medium (i.e. reduced CM medium lacking Tyr), and used to inoculate two separate pulse-feed alternating turbidostats at an initial OD_600_ of 1.46. After 45 days of continuous growth under selective conditions (in the absence of Tyr, Phe and tetracycline), four and five clones from the individual cultures were sequenced and analyzed by restriction digestion of their CM-encoding plasmids (Figure S7).

### Molecular dynamics simulations

Dimeric models of the reduced-alphabet CMs were created by homology-modeling, based on the EcCM X-ray structure [27], followed by *in silico* mutagenesis to generate 10-CM and 11-CM from 9-CM. One TSA molecule was added to each active site. All structures were first energy-minimized using the GROMOS 53A6 force field [38], [39] prior to further analysis. Molecular dynamics (MD) simulations of each protein/ligand system in SPC water were run using the GROMOS05 software [40] under NpT conditions with a pressure of 1 atm and a temperature of 293 K. All analyses were carried out using the GROMOS++ suite of programs [41].

## Supporting Information

Figure S1 Relative levels of CM production. Differential expression of CM constructs was analyzed in lysates from *E. coli* grown in the presence of 1000 ng/mL tetracycline by western blot using anti-His antibodies conjugated to HRP. Lanes 1–3 correspond to 9-, 10-, and 11-CM encoded on the high-copy plasmid pKT. For comparison, lysates from cells with chromosomally encoded 9-CM (lane 4), the plasmid-encoded AT-rich gene (lane 5), an empty vector (lane 6), and the wild-type CM from *E. coli* (lane 7), were included in the analysis. The low signal of EcCM might be due to epitope occlusion, or fast CM degradation, tolerated by the fact that only very low concentrations of EcCM suffice to confer growth in the absence of aromatic amino acids.
(TIF)

Figure S2 Detailed view of amino acid substitutions at mutation hot spots for cassette mutagenesis libraries. The fractions of complementing clones are plotted for each combination of residues at positions 30 and 61 in the presence of L-Phe and L-Tyr (A, unselected library), and in the absence of aromatic amino acids with CM variants induced with 1000 ng/mL (B) and 100 ng/mL (C) tetracycline. X denotes any of the proteinogenic amino acids not individually listed.
(TIF)

Figure S3 *In vivo* complementation of CM constructs. (A) Comparison of 9-CM and its evolved variants (9-CM: red, 10-CM: blue, 11-CM: black), along with a positive (EcCM, green) and a negative control (CM with a frameshift, yellow). (B) Selected variants from cassette mutagenesis, referred to according to their mutations, in comparison to 11-CM (I30T/L61V). The number in parenthesis indicates at which concentration of tetracycline the mutants were picked from in the original selection experiment. CM mutant genes are expressed from plasmid pKT in KA12/pKIMP-UAUC. Growth is rated according to an arbitrary scale (Table S5) of colony sizes from single colony streak-outs on M9c agar plates [45] after 3 days at 30°C (note that the rating of 0–2 indicates that there is no single colony growth yet but just cell material visible on the plates). Variation of the selection stringency was achieved through changes in tetracycline concentration. The indicator “+FY” denotes growth experiments performed in the presence of Phe and Tyr in the medium.
(TIF)

Figure S4 Biophysical characterization of 9-CM and evolved variants. (A) Size-exclusion chromatography of CM variants. The dimer peak (major peak around 170 mL) was isolated for each sample. Red: 9-CM, blue: 10-CM, black: 11-CM. (B) CD spectra of 16 µM CM. (C) Thermal denaturation (bottom) and renaturation (top) curves determined by monitoring CD at 222 nm.
(TIF)

Figure S5 Limited proteolysis. (A) SDS PAGE analysis. CM variants were incubated for the indicated period with trypsin under native conditions to assay protein compactness. (B) LC-MS analysis of the trypsin cleavage of 11-CM after 60 min. The main proteolysis fragment corresponds to 11-CM(1–92) (calculated mass = 11448.7), side products include 11-CM(1–91) (calculated mass = 11320.5) and 11-CM(1–87) (calculated mass = 10818.8).
(TIF)

Figure S6 Indicators of the structural stability of the CM variants during the 10 ns MD simulation. The top panel shows the atom-positional rmsd of the Cα atoms, calculated individually for each subunit after aligning the structure to the energy-minimized reference state of that subunit by minimizing the rmsd of all Cα atoms. The lower four panels depict CM secondary structure content. The colors used for the different CM variants and types of secondary structure are indicated in the boxes to the right.
(TIF)

Figure S7 Analysis of plasmid recombination from long-term evolution of AT9-cm. Plasmid maps of the helper plasmid pKIMP-UAUC, pKECMT-AT9-cm, and the resulting recombined plasmid denoted pKIMP/AT9-cm are shown on top. Below, the size of the plasmids is given, followed by the (presumed) coordinates of some restriction sites and the expected sizes of digested fragments. An agarose gel of a restriction digestion of pKIMP/AT9-cm is displayed at the bottom of the figure.
(TIF)

Figure S8 Characterization of chromosomal constructs by PCR and restriction analysis. (A) Map of the chromosomal region surrounding *CM* genes upon recombination into the *kdgK* locus of the KA12 genome. (B) Scheme of PCR and restriction digestion analysis to verify the location and orientation of the inserted CM cassettes. (C) Agarose gel of the analysis described in (B).
(TIF)

Table S1 Codon usage of genes *n9-cm* and *AT9-cm* coding for 9-CM.
(DOCX)

Table S2 Sequences of members of a library from cassette mutagenesis at positions 30 and 61 that were obtained under non-selective conditions.
(DOCX)

Table S3 Sequences of complementing members of a library from cassette mutagenesis at positions 30 and 61.
(DOCX)

Table S4 Composition of CM-medium used for CM selection in turbidostats.
(DOCX)

Table S5 Growth scale for *in vivo* complementation assays.
(DOCX)

Text S1 Detailed experimental procedures.
(DOCX)
