# Supplementary figures and images for: Directed Evolution of a Model Primordial Enzyme Provides Insights into the Development of the Genetic Code
Source: PLoS Genet. 2013 Jan 3;9(1):e1003187. doi: 10.1371/journal.pgen.1003187 (PMC3536711; doi:10.1371/journal.pgen.1003187)

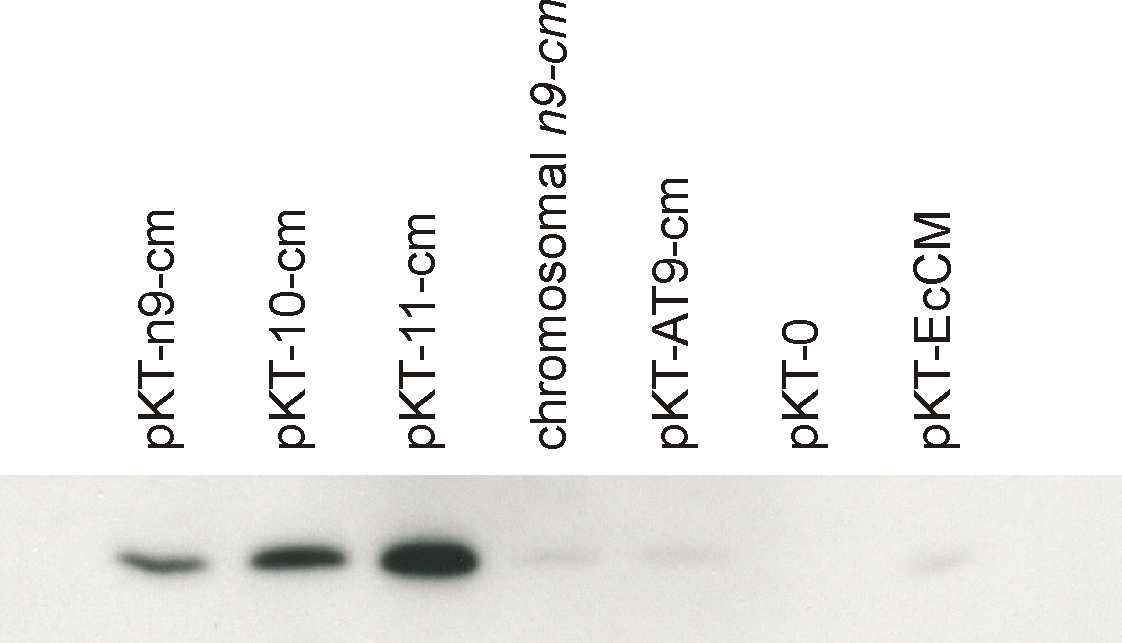

Supplement: Figure S1 — Relative levels of CM production. Differential expression of CM constructs was analyzed in lysates from E. coli grown in the presence of 1000 ng/mL tetracycline by western blot using anti-His antibodies conjugated to HRP. Lanes 1–3 correspond to 9-, 10-, and 11-CM encoded on the high-copy plasmid pKT. For comparison, lysates from cells with chromosomally encoded 9-CM (lane 4), the plasmid-encoded AT-rich gene (lane 5), an empty vector (lane 6), and the wild-type CM from E. coli (lane 7), were included in the analysis. The low signal of EcCM might be due to epitope occlusion, or fast CM degradation, tolerated by the fact that only very low concentrations of EcCM suffice to confer growth in the absence of aromatic amino acids. (TIF) [file pgen.1003187.s001.tif]

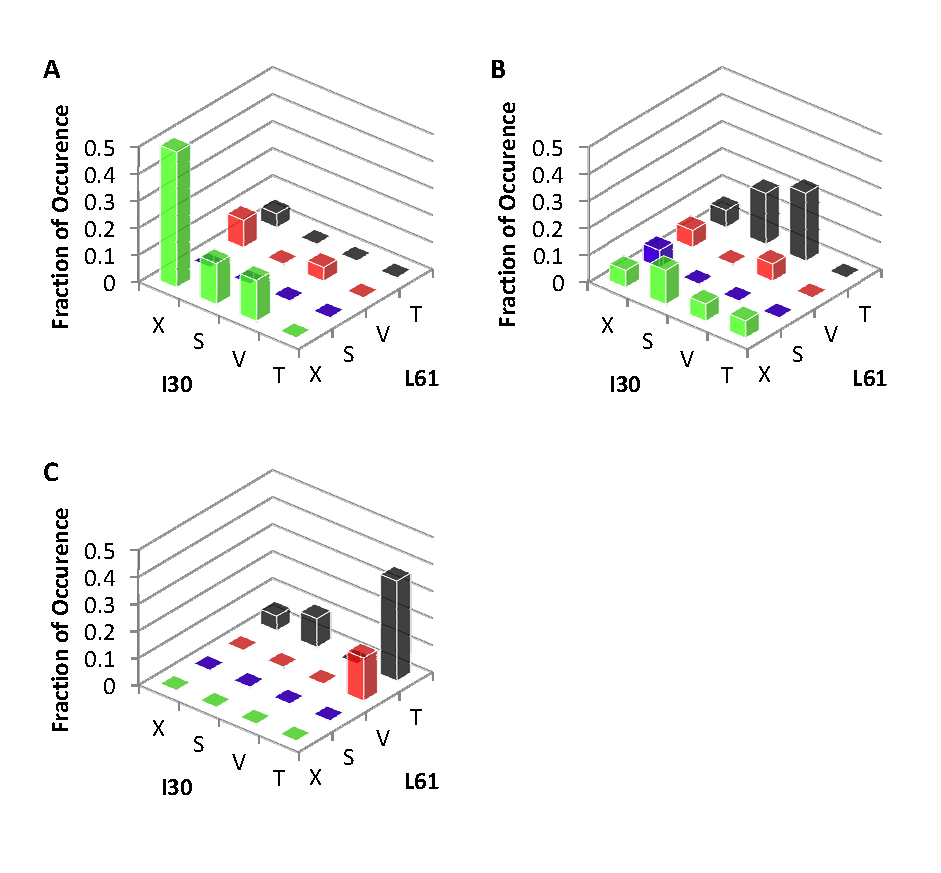

Supplement: Figure S2 — Detailed view of amino acid substitutions at mutation hot spots for cassette mutagenesis libraries. The fractions of complementing clones are plotted for each combination of residues at positions 30 and 61 in the presence of L-Phe and L-Tyr (A, unselected library), and in the absence of aromatic amino acids with CM variants induced with 1000 ng/mL (B) and 100 ng/mL (C) tetracycline. X denotes any of the proteinogenic amino acids not individually listed. (TIF) [file pgen.1003187.s002.tif]

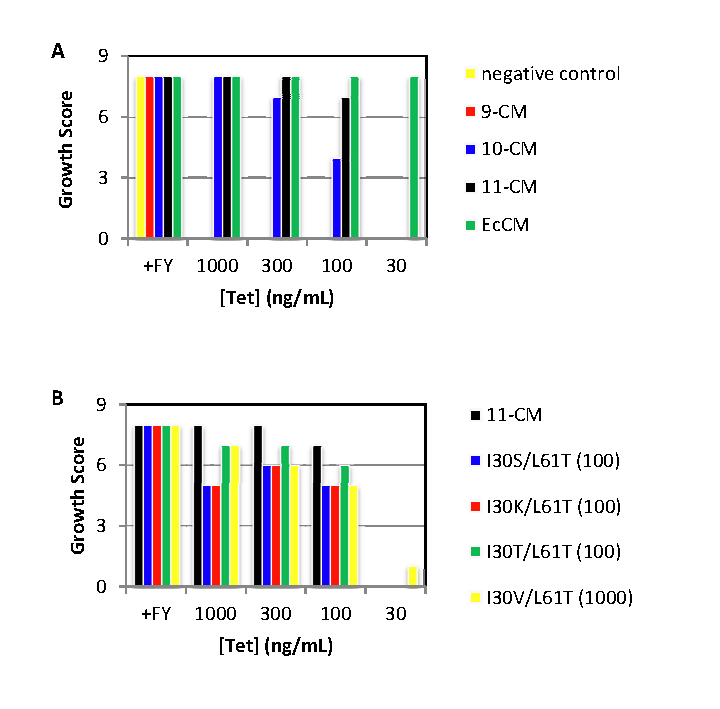

Supplement: Figure S3 — In vivo complementation of CM constructs. (A) Comparison of 9-CM and its evolved variants (9-CM: red, 10-CM: blue, 11-CM: black), along with a positive (EcCM, green) and a negative control (CM with a frameshift, yellow). (B) Selected variants from cassette mutagenesis, referred to according to their mutations, in comparison to 11-CM (I30T/L61V). The number in parenthesis indicates at which concentration of tetracycline the mutants were picked from in the original selection experiment. CM mutant genes are expressed from plasmid pKT in KA12/pKIMP-UAUC. Growth is rated according to an arbitrary scale (Table S5) of colony sizes from single colony streak-outs on M9c agar plates [45] after 3 days at 30°C (note that the rating of 0–2 indicates that there is no single colony growth yet but just cell material visible on the plates). Variation of the selection stringency was achieved through changes in tetracycline concentration. The indicator “+FY” denotes growth experiments performed in the presence of Phe and Tyr in the medium. (TIF) [file pgen.1003187.s003.tif]

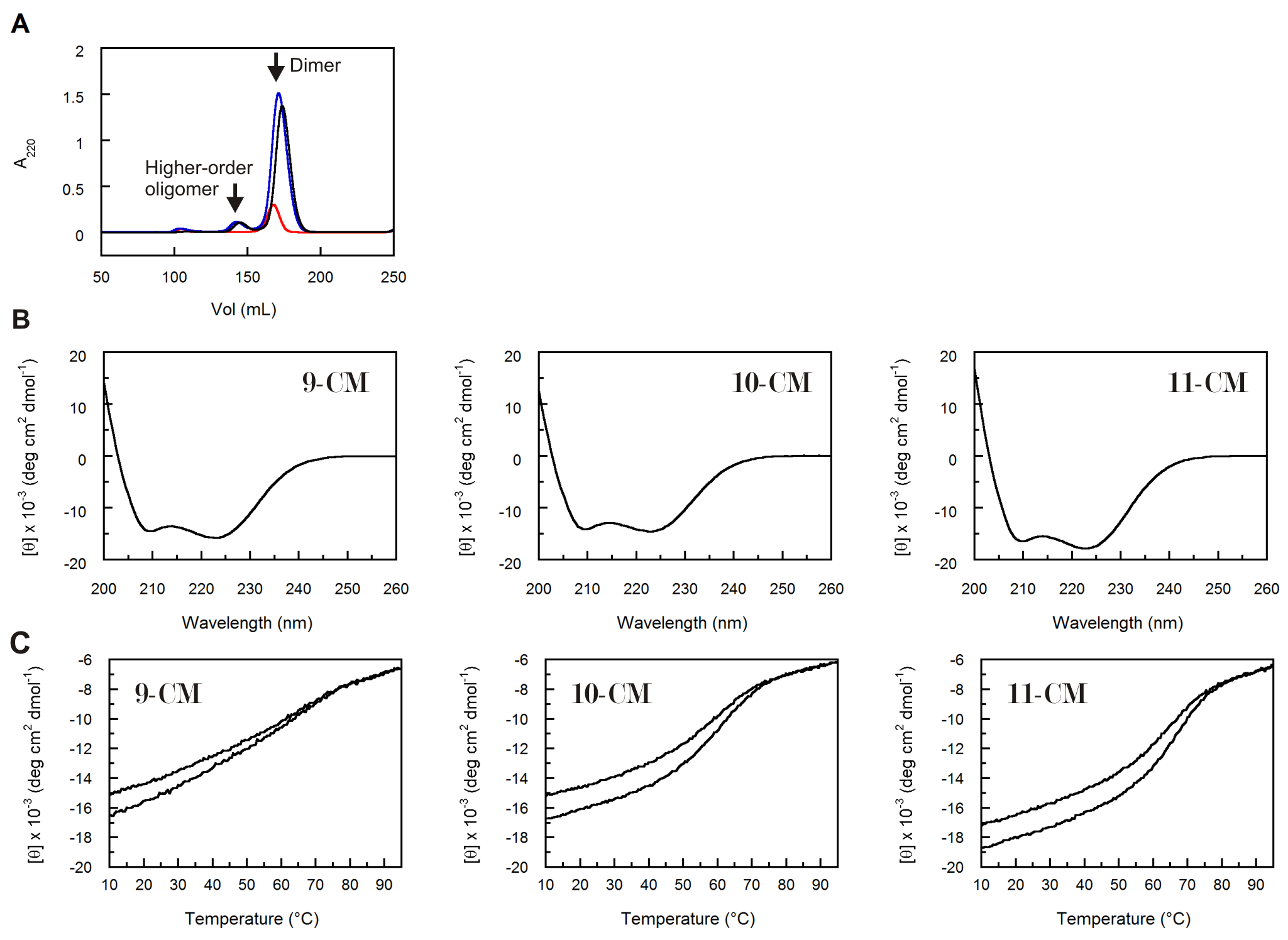

Supplement: Figure S4 — Biophysical characterization of 9-CM and evolved variants. (A) Size-exclusion chromatography of CM variants. The dimer peak (major peak around 170 mL) was isolated for each sample. Red: 9-CM, blue: 10-CM, black: 11-CM. (B) CD spectra of 16 µM CM. (C) Thermal denaturation (bottom) and renaturation (top) curves determined by monitoring CD at 222 nm. (TIF) [file pgen.1003187.s004.tif]

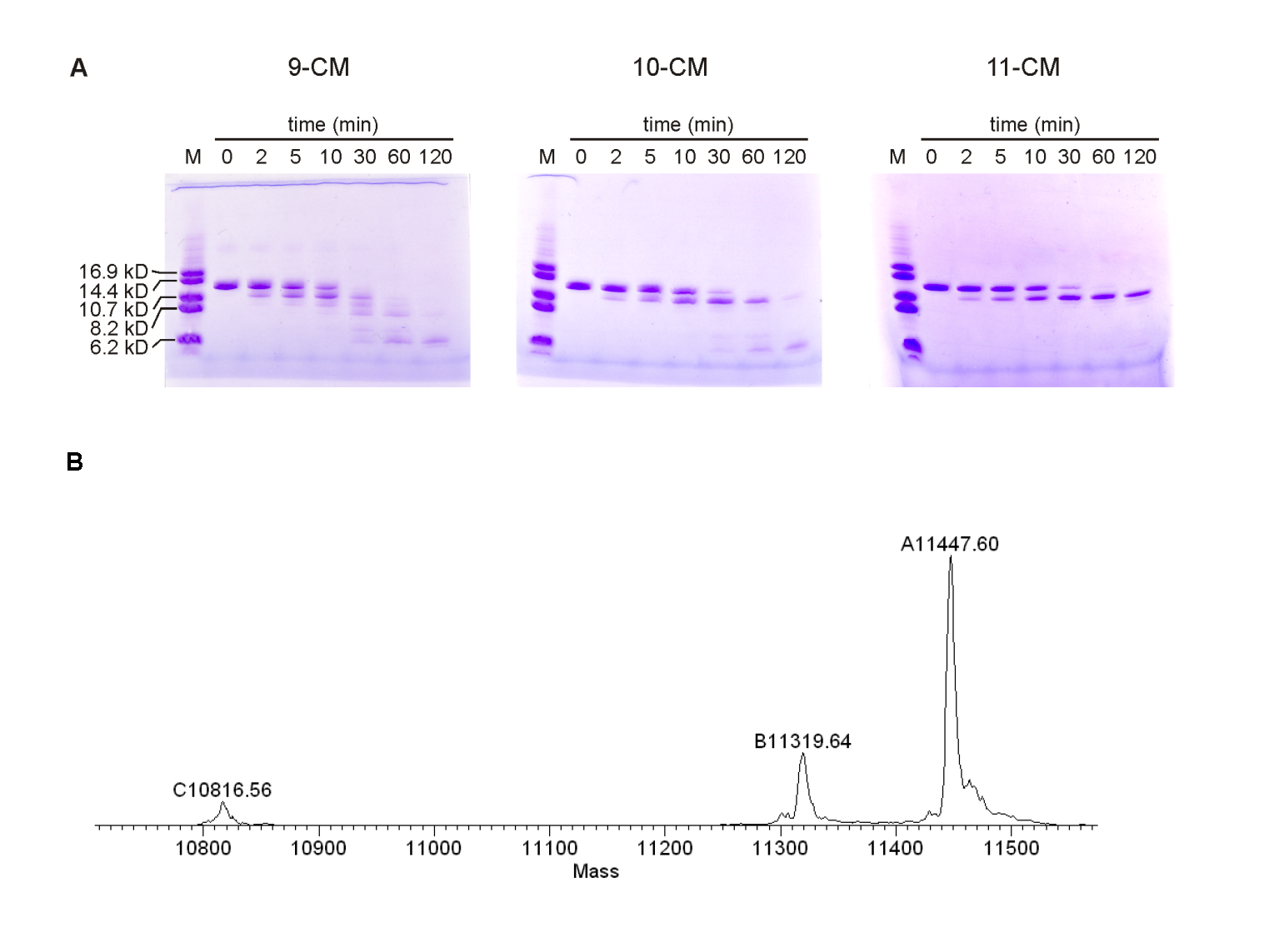

Supplement: Figure S5 — Limited proteolysis. (A) SDS PAGE analysis. CM variants were incubated for the indicated period with trypsin under native conditions to assay protein compactness. (B) LC-MS analysis of the trypsin cleavage of 11-CM after 60 min. The main proteolysis fragment corresponds to 11-CM(1–92) (calculated mass = 11448.7), side products include 11-CM(1–91) (calculated mass = 11320.5) and 11-CM(1–87) (calculated mass = 10818.8). (TIF) [file pgen.1003187.s005.tif]

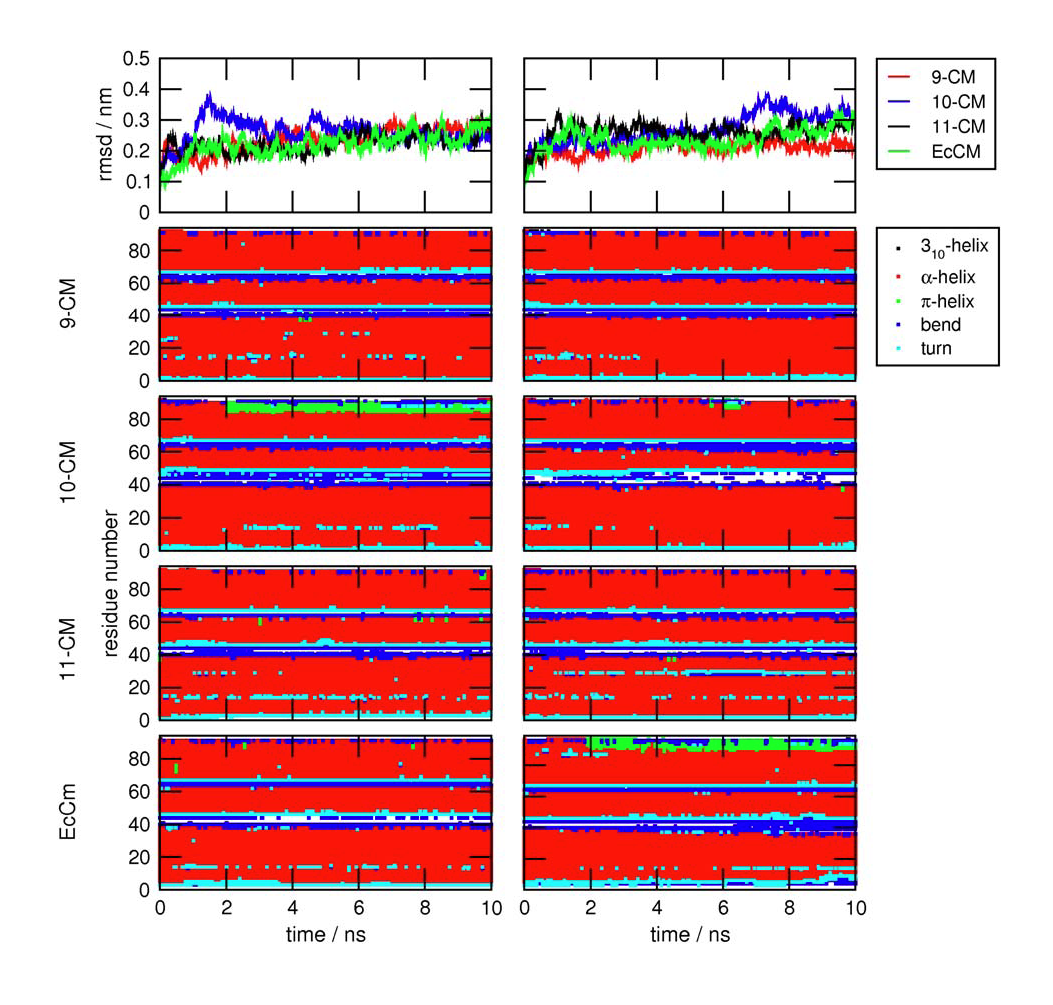

Supplement: Figure S6 — Indicators of the structural stability of the CM variants during the 10 ns MD simulation. The top panel shows the atom-positional rmsd of the Cα atoms, calculated individually for each subunit after aligning the structure to the energy-minimized reference state of that subunit by minimizing the rmsd of all Cα atoms. The lower four panels depict CM secondary structure content. The colors used for the different CM variants and types of secondary structure are indicated in the boxes to the right. (TIF) [file pgen.1003187.s006.tif]

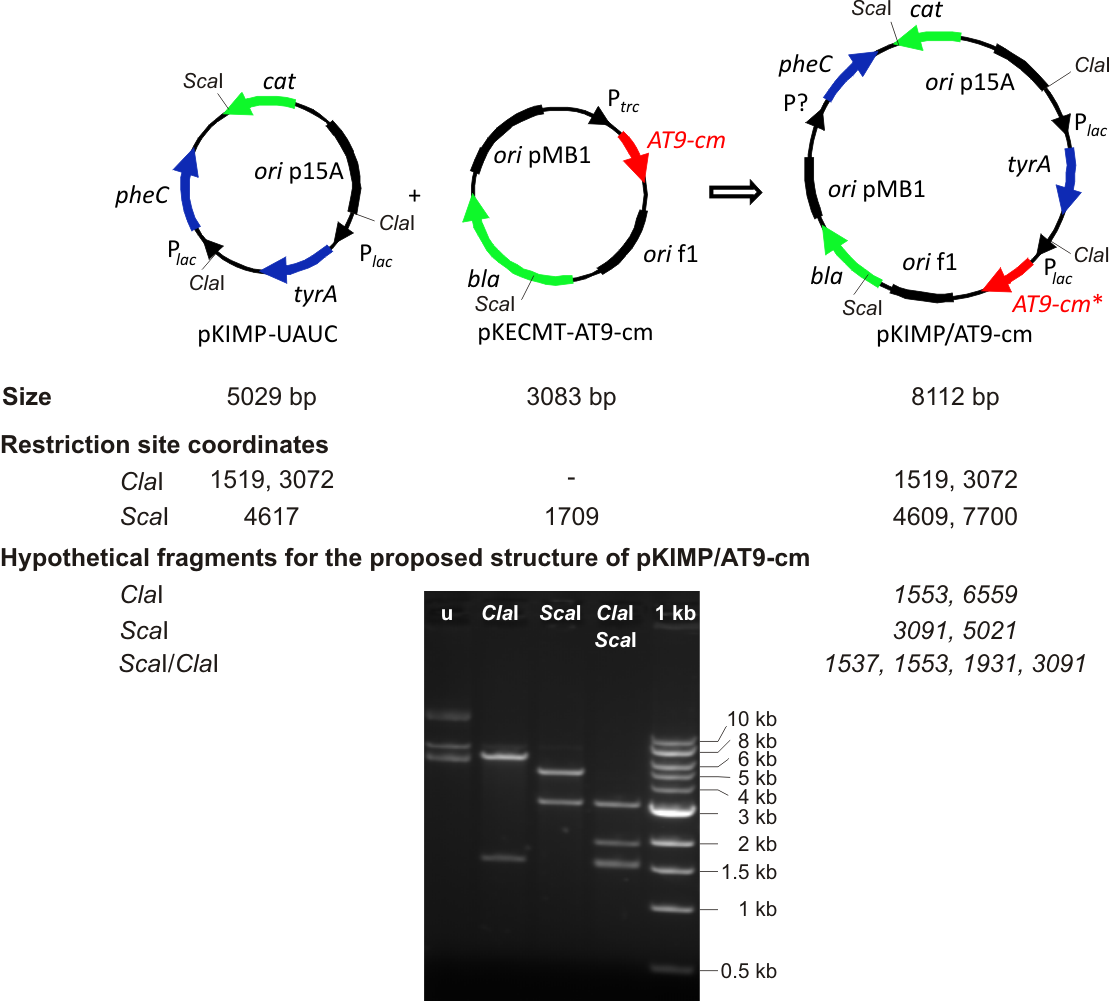

Supplement: Figure S7 — Analysis of plasmid recombination from long-term evolution of AT9-cm. Plasmid maps of the helper plasmid pKIMP-UAUC, pKECMT-AT9-cm, and the resulting recombined plasmid denoted pKIMP/AT9-cm are shown on top. Below, the size of the plasmids is given, followed by the (presumed) coordinates of some restriction sites and the expected sizes of digested fragments. An agarose gel of a restriction digestion of pKIMP/AT9-cm is displayed at the bottom of the figure. (TIF) [file pgen.1003187.s007.tif]

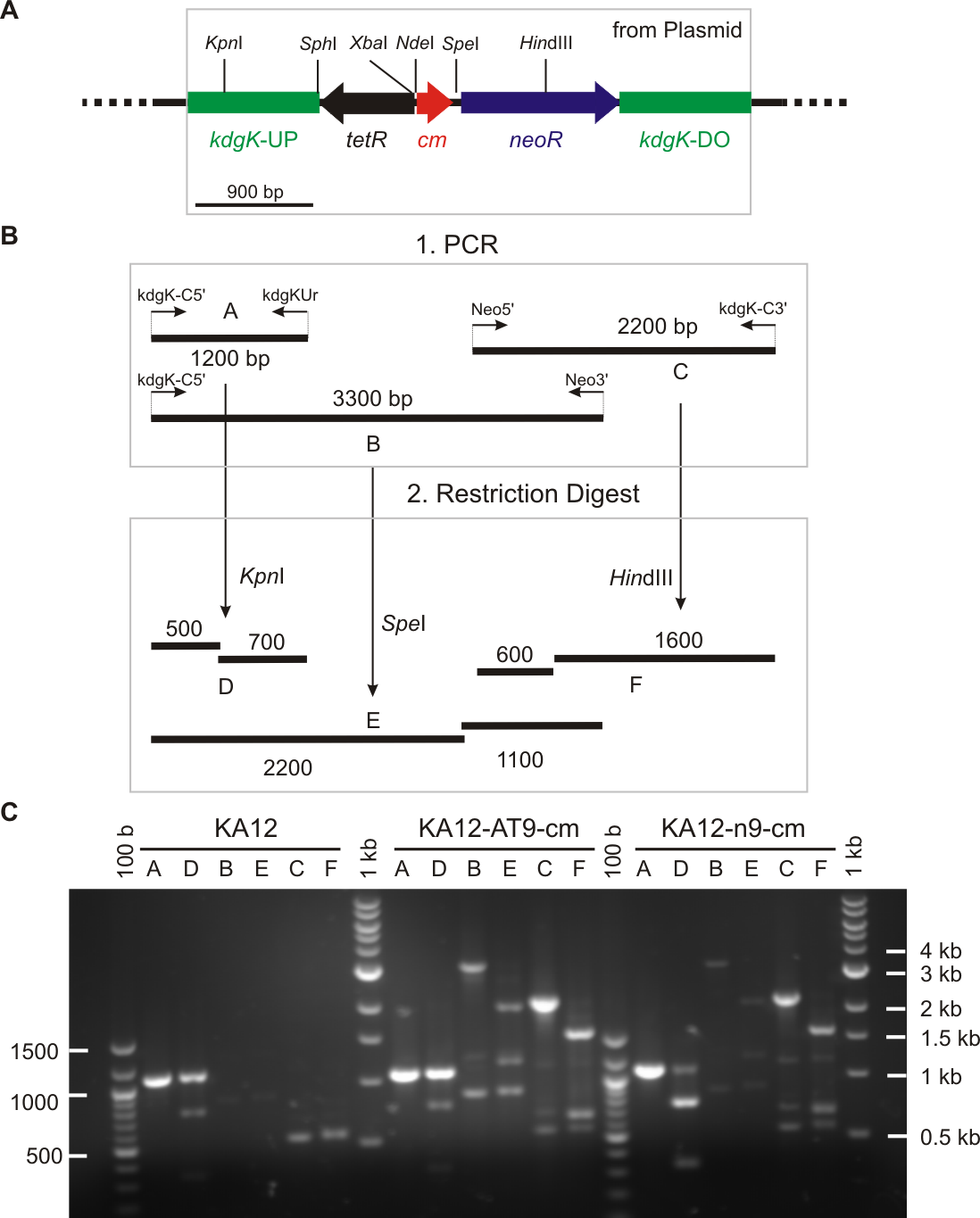

Supplement: Figure S8 — Characterization of chromosomal constructs by PCR and restriction analysis. (A) Map of the chromosomal region surrounding CM genes upon recombination into the kdgK locus of the KA12 genome. (B) Scheme of PCR and restriction digestion analysis to verify the location and orientation of the inserted CM cassettes. (C) Agarose gel of the analysis described in (B). (TIF) [file pgen.1003187.s008.tif]
