# Supplementary material for: Directed Evolution of a Model Primordial Enzyme Provides Insights into the Development of the Genetic Code
Source: PLoS Genet. 2013 Jan 3;9(1):e1003187. doi: 10.1371/journal.pgen.1003187 (PMC3536711; doi:10.1371/journal.pgen.1003187)
Supplement: Table S1 — Codon usage of genes n9-cm and AT9-cm coding for 9-CM. (DOCX) [file pgen.1003187.s009.docx]

**Table S1**: Codon usage of genes *n9-cm* and *AT9-cm* coding for 9-CM.

| **AA** | **Codon** | ***n9-cm*** | ***AT9-cm*** |
| --- | --- | --- | --- |
| Phe | UUU |  | 9 |
|  | UUC | 9 |  |
| Leu | UUA |  | 16 |
|  | UUG | 6 |  |
|  | CUU | 1 |  |
|  | CUC | 4 |  |
|  | CUG | 5 |  |
| Ile | AUU |  | 9 |
|  | AUC | 9 |  |
| Met | AUG | 6 | 6 |
| Asn | AAU |  | 8 |
|  | AAC | 8 |  |
| Lys | AAA | 13 | 20 |
|  | AAG | 7 |  |
| Asp | GAU |  | 10 |
|  | GAC | 10 |  |
| Glu | GAA | 8 | 12 |
|  | GAG | 4 |  |
| Arg | CGU | 3 |  |
|  | AGA |  | 3 |
| Sum |  | 93 | 93 |
