# Supplementary material for: Directed Evolution of a Model Primordial Enzyme Provides Insights into the Development of the Genetic Code
Source: PLoS Genet. 2013 Jan 3;9(1):e1003187. doi: 10.1371/journal.pgen.1003187 (PMC3536711; doi:10.1371/journal.pgen.1003187)
Supplement: Table S2 — Sequences of members of a library from cassette mutagenesis at positions 30 and 61 that were obtained under non-selective conditions. (DOCX) [file pgen.1003187.s010.docx]

**Table S2:** Sequences of members of a library from cassette mutage­nesis at positions 30 and 61 that were obtained under non-selective conditions.

|  | **Residue 30** | |  | **Residue 61** | |
| --- | --- | --- | --- | --- | --- |
| **clone** | **DNA** | **Protein** |  | **DNA** | **Protein** |
| G1 | CTC | Leu |  | AGG | Arg |
| G2 | ATC | Ile |  | TTG | Leu |
| G3 | TAC | Tyr |  | CTC | Leu |
| G4 | GTG | Val |  | CTC | Leu |
| G5 | TTC | Phe |  | GTC | Val |
| G6 | GTC | Val |  | ATC | Ile |
| G8 | TGG | Trp |  | CCG | Pro |
| G9 | CCG | Pro |  | TAG | Stop |
| G11 | TAG | Stop |  | CAC | His |
| G12 | ATC | Ile |  | AAC | Asn |
| H1 | GTC | Val |  | CAC | His |
| H3 | ATC | Ile |  | AAC | Asn |
| H4 | TGC | Cys |  | CAC | His |
| H5 | TCC | Ser |  | CTG | Leu |
| H6 | CTG | Leu |  | GTC | Val |
| H7 | TCG | Ser |  | AAC | Asn |
| H8 | CGG | Arg |  | AAC | Asn |
| H9 | AGC | Ser |  | ATG | Met |
| H10 | GCG | Ala |  | ACG | Thr |
| H11 | GTC | Val |  | GTC | Val |
