# Supplementary material for: Directed Evolution of a Model Primordial Enzyme Provides Insights into the Development of the Genetic Code
Source: PLoS Genet. 2013 Jan 3;9(1):e1003187. doi: 10.1371/journal.pgen.1003187 (PMC3536711; doi:10.1371/journal.pgen.1003187)
Supplement: Table S3 — Sequences of complementing members of a library from cassette mutagenesis at positions 30 and 61. (DOCX) [file pgen.1003187.s011.docx]

**Table S3:** Sequences of complementing members of a library from cassette mutagenesis at positions 30 and 61.

| **[Tet]** |  | **Residue 30** | |  | **Residue 61** | |
| --- | --- | --- | --- | --- | --- | --- |
| **(ng/mL)** | **clone** | **DNA** | **Protein** |  | **DNA** | **Protein** |
| 1000 | D11 | GTG | Val |  | ACG | Thr |
| 1000 | E2 | GCG | Ala |  | TCG | Ser |
| 1000 | E3 | GTG | Val |  | ACG | Thr |
| 1000 | E4 | AGC | Ser |  | GGC | Gly |
| 1000 | E5 | GTG | Val |  | GTG | Val |
| 1000 | E6 | ACC | Thr |  | ATG | Met |
| 1000 | E10 | GTC | Val |  | ACC | Thr |
| 1000 | E11 | TCC | Ser |  | ACG | Thr |
| 1000 | E12 | TGC | Cys |  | ACC | Thr |
| 1000 | F2 | TCG | Ser |  | ACG | Thr |
| 1000 | F3 | GTC | Val |  | ACG | Thr |
| 1000 | F4 | GCC | Ala |  | GTG | Val |
| 1000 | F5 | GGG | Gly |  | GCG | Ala |
| 1000 | F10 | GTC | Val |  | TTC | Phe |
| 1000 | F11 | TCG | Ser |  | ACC | Thr |
| 1000 | F12 | AGC | Ser |  | TGC | Cys |
| 100 | B3 | ACG | Thr |  | GTG | Val |
| 100 | B4 | ACG | Thr |  | GTG | Val |
| 100 | B8 | ACC | Thr |  | ACC | Thr |
| 100 | B11 | ACG | Thr |  | ACC | Thr |
| 100 | B12 | ACG | Thr |  | ACG | Thr |
| 100 | C2 | TCC | Ser |  | ACG | Thr |
| 100 | C4 | ACG | Thr |  | ACG | Thr |
| 100 | C5 | AAG | Lys |  | ACC | Thr |
| 100 | C6 | ACC | Thr |  | ACG | Thr |
| 100 | D1 | ACG | Thr |  | GTG | Val |
| 100 | D3 | ACG | Thr |  | ACC | Thr |
| 100 | D5 | AGC | Ser |  | ACG | Thr |
| 100 | D6 | ACG | Thr |  | ACG | Thr |
