# Supplementary material for: Directed Evolution of a Model Primordial Enzyme Provides Insights into the Development of the Genetic Code
Source: PLoS Genet. 2013 Jan 3;9(1):e1003187. doi: 10.1371/journal.pgen.1003187 (PMC3536711; doi:10.1371/journal.pgen.1003187)
Supplement: Table S4 — Composition of CM-medium used for CM selection in turbidostats. (DOCX) [file pgen.1003187.s012.docx]

**Table S4:** Composition of CM-medium used for CM selection in turbidostats. Components marked by * are omitted in “reduced CM medium”. Reduced CM medium also lacking L-Phe is designated as “‑Aro”. Additives in italics are included only when indicated in the text.

| **Basis Medium** | **pH 7.0** |  | **NTA mix** | **pH 6.5** |
| --- | --- | --- | --- | --- |
| Citric Acid | 4 mM |  | Nitrilotriacetic acid | 10 mM |
| MgSO_4_ | 1 mM |  | CaCl_2_ | 3 mM |
| NH_4_Cl | 20 mM |  | FeCl_3_ | 3 mM |
| K_2_HPO_4_ | 50 mM |  | MnCl_2_ | 1 mM |
| D(+)Glucose | 0.2% w/v |  | ZnCl_2_ | 0.3 mM |
| NTA mix | 1:1000 |  | H_3_BO_3_ | 0.3 mM |
| 4-Hydroxybenzoic acid* | 5 mg/mL |  | CrCl_3_ | 0.3 mM |
| 4-Aminobenzoic acid* | 5 mg/mL |  | CoCl_2_ | 0.3 mM |
| 2,3-Dihydroxybezoic acid* | 1.55 mg/mL |  | CuCl_2_ | 0.3 mM |
| L-Tryptophan* | 20 mg/mL |  | NiCl_2_ | 0.3 mM |
| Thiamine HCl | 5 mg/mL |  | Na_2_MoO_4_ | 0.3 mM |
| *L-Phenylalanine* | 121 μM |  | Na_2_SeO_3_ | 0.3 mM |
| *Tetracycline* | 4.5 μM |  |  |  |
