# Supplementary material for: Directed Evolution of a Model Primordial Enzyme Provides Insights into the Development of the Genetic Code
Source: PLoS Genet. 2013 Jan 3;9(1):e1003187. doi: 10.1371/journal.pgen.1003187 (PMC3536711; doi:10.1371/journal.pgen.1003187)
Supplement: Table S5 — Growth scale for in vivo complementation assays. (DOCX) [file pgen.1003187.s013.docx]

**Table S5:** Growth scale for *in vivo* complementation assays.

| **Size Scale** | **Assigned Value** |
| --- | --- |
| No visible growth | 0 |
| Some cell material at start of streak-out | 1 |
| Cell material in “first dimension” | 2 |
| Single colonies visible as dots: with open lid only | 3 |
| Single colonies visible as dots: against light | 4 |
| Small single colonies, but immediately visible | 5 |
| Small single colonies that are easy to pick | 6 |
| Middle-sized single colonies | 7 |
| Large single colonies | 8 |
| Giant single colonies | 9 |
