# Supplementary material for: Directed Evolution of a Model Primordial Enzyme Provides Insights into the Development of the Genetic Code
Source: PLoS Genet. 2013 Jan 3;9(1):e1003187. doi: 10.1371/journal.pgen.1003187 (PMC3536711; doi:10.1371/journal.pgen.1003187)
Supplement: Text S1 — Detailed experimental procedures. (DOCX) [file pgen.1003187.s014.docx]

## Text S1 – Detailed Experimental Procedures

### *E. coli* strains

*E. coli* strains KA12 (genotype: ∆(*srlR-recA*)306:Tn*10*, ∆(*pheA-tyrA-aroF*), *thi-1, endA-1, hsdR17*, ∆(*argF-lac*)*U169, supE44*) [1,2] and XL1-Blue (Strata­gene) were used for cloning purposes. KA13, a CM-deficient expression strain [3], was used for protein production, and KA12/pKIMP-UAUC [1] for *in vivo* selection experiments.

### Molecular cloning

General cloning procedures followed standard protocols [4]. DNA concen­trations were determined using a NanoDrop spectrophotometer (Witec AG). For initial selection experiments, the *n9-cm* gene was subcloned as a 282 bp *Nde*I-*Xho*I fragment from a pET-22b-pATCH [5] derivative containing the simplified 9-amino acid CM [6] gene into the 4561 bp *Nde*I-*Xho*I fragment of pMG211[7] (pMG211-n9-cm). The *AT9-cm* gene was synthesized by Microsynth, and ligated with pMG211 (pMG211-AT9-cm) analogously to the normal codon gene. For both genes the 282 bp *Nde*I-*Xho*I fragments were also ligated with the 3338 bp *Nde*I-*Xho*I fragment of pKT [8] and the 2801 bp *Nde*I-*Xho*I fragment of pKECMT [9] to yield plasmids pKT-n9cm, pKT-AT9-cm, pKECMT-n9cm, and pKECMT-AT9-cm, respectively. Se­quencing of pMG211, pKT and pKECMT constructs was performed using the BigDye v3.1 kit and analysis on a 3100-Avant genetic analyzer (Applied Biosys­tems) with primers T7, T7TR, pKTsel-seq, or M13-21 (**see Table below**). For library sequencing, plasmid preparations were performed with the Wizard MagneSil kit (Promega) in a 96-well format. For direct sequencing of CM variants encoded on the *E. coli* chromosome, CM genes were first ampliﬁed by colony PCR (primers pKtCMseq and pKtCMu2), then purified with a NucleoSpin PCR purification kit (Machery Nagel), and ﬁnally sequenced (primer pKtCMseq). For each variant, the PCR product of one clone was digested with *Nde*I and *Xho*I (282 bp) and ligated with the 4560 bp *Nde*I-*Xho*I fragment of pMG211 for protein production. All primers, synthesized and puriﬁed by Microsynth, are listed below.

**Primer sequences:**

| Primer | Sequence |
| --- | --- |
| T7 | TAATACGACTCACTATAGG |
| T7TR | AAGACCCGTTTAGAGGCCCCAA |
| pKTsel-seq | TGTAAAACGACGGCCAG |
| M13-21 | CCACAACGGTTTCCCTCT |
| pKtCMseq | CCAGCAAACCACCACAGCGCAAACTAACG |
| pKtCMu2 | CAGAGATTTTGAGACACAACGT |
| kdgKUf | AAATGGGATCCCTTATAGCGCCAGAACCGCC |
| kdgKUr | CTCCTCTTTAATAAAGCAAATAAATTTTTTATGATTTGCATGCAGTCGTGAACCTGATCTGTAAAAA |
| kdgKDf | CGCAGGAATTCCAAAAGGCTGGATAAAGTGTTACC |
| kdgKDr | ATCCTCCGCGGCCGCTGAAACAACCCGTTGA |
| cassetter | CTTTCCGGCCGAGCTGACTAGTCATTAAGATCTTATTTCATATGTTTCTCCTCTTTAATAAAGCAAATAA |
| kdgK-C5’ | GCCAGTCCTAAAGATAGTCC |
| kdgK-C3’ | GTTAGGTCACGATCCTGCCGAT |
| Neo5’ | GTTGTGTCTCAAAATCTCTGATG |
| Neo3’ | GACTCATACCAGGCCTGAATCG |
| 30r | GAGCTTGTTACGCTTCAGG |
| 30fx | CCTGAAGCGTAACAAGCTC*NNS*AACGAAATCTTCAAACTC |
| 61rx | CGTCCATGTTGTTCTCCTT*SNN*GAACTTTTTGATTTTGTCCAG |
| 61f | AAGGAGAACAACATGGACG |

### DNA sequences

The DNA sequences encoding the simplified mutases are given below.

*n9-cm*

1 ATGTTGAAAG ACATCATGAA GTTGCGTAAC AAAATCGACG AGCTGGACGA GGACCTGTTG

61 AAGCTTTTCC TGAAGCGTAA CAAGCTCATC AACGAAATCT TCAAACTCAA GAAAGAACTG

121 AACATGGACT TGAACGACTT CGACCGTGAA GAGTTCATCC TGGACAAAAT CAAAAAGTTC

181 TTGAAGGAGA ACAACATGGA CGAAGACCTC TTCATCAAAA TCTTCAAATT CTTGTTCGAA

241 AAAAACAAAA TGATCGAAAA AGAAATGCTC AAAAAAGAAC TCGAGCACCA CCACCACCAC

301 CACTAATAAT GA

*AT9-cm*

1 ATGTTAAAAG ATATTATGAA ATTAAGAAAT AAAATTGATG AATTAGATGA AGATTTATTA

61 AAATTATTTT TAAAAAGAAA TAAATTAATT AATGAAATTT TTAAATTAAA AAAAGAATTA

121 AATATGGATT TAAATGATTT TGATAGAGAA GAATTTATTT TAGATAAAAT TAAAAAATTT

181 TTAAAAGAAA ATAATATGGA TGAAGATTTA TTTATTAAAA TTTTTAAATT TTTATTTGAA

241 AAAAATAAAA TGATTGAAAA AGAAATGTTA AAAAAAGAAC TCGAGCACCA CCACCACCAC

301 CACTAATAAT GA

The DNA sequences of the C-terminal hexa-histidine tag, separated from the protein by a Leu-Glu dipeptide (encoded by the *Xho*I site) is indicated in red. The two sequences, which encode identical proteins, differ by almost 30% in their nucleotide sequence. The difference in codon usage is indicated in **Table S1** (only the CM portion of the sequence is included). The genes use different codons at 70% of the positions, which results in a 20% difference in amino acid substitutions accessible by point mutation.

### Construction of *E. coli* strains harboring a chromosomal copy of 9-CM encoding genes

The construction of strains expressing CM genes chromosomally was performed according to Gamper and Kast [10]. The vector for genome targeting was assem­bled in a 4-fragment ligation as follows. The 10427-bp plasmid pMG207 [10] was digested with *Sac*II and *Bam*HI to give three fragments: The **5104** bp fragment carrying *recA* (for homologous recombination in RecA^-^ strains), *bla* (antibiotic selection marker) and a mu­tant *pheS* (conditional lethality selection marker) was treated with *Stu*I to remove potential contaminations, puriﬁed by gel electrophoresis, and used for the ligation. The 4225 bp fragment of pMG207 was further digested with *Eco*RI and *Eag*I, and the resulting **1208** bp fragment, carrying *agp* (which confers kanamycin resistance) was isolated for the ligation. The third fragment (1098 bp) was discarded. The upstream and downstream chromosomal flanking regions needed for homologous recombination were amplified by PCR with Phusion polymerase (Finzymes) from genomic DNA of KA12 using primer pairs kdgKUf / kdgKUr (1054 bp), and kdgKDf / kdgKDr (1021 bp), respectively. The upstream frag­ment was furnished with a cloning site by PCR with Taq polymerase using primers kdgKUf and cassetter (1101 bp), and digested with *Bam*HI and *Eag*I, yielding a **1085** bp fragment. The downstream fragment was directly digested with *Sac*II and *Eco*RI, yielding a **1005** bp fragment. Approximately 100 ng of each of the 1208 bp (*agp* from pMG207), 1085 bp (*kdgK* upstream) and 1005 bp (*kdgK* downstream) fragments and 300 ng of the 5104 bp fragment (pMG207 backbone) were ligated overnight at 16 °C with 2000 U T4 DNA ligase yielding pKtCM. Correct assembly of the fragments was verified by PCR and restriction digestions according to previously published procedures [10].

CM variants and the P*_tet_* promoter were inserted into the pKtCM plasmid ac­cording to the following procedure. First, the 314 bp *Nde*I-*Spe*I fragments from pMG211-n9cm and pMG211-AT9-CM were ligated into *Nde*I- and *Spe*I-digested pKtCM (8382 bp fragment). The resulting plasmids (8696 bp) were subsequently digested with *Sph*I and *Nde*I (8650 bp), and fused with the *tetR* gene, excised from pAC-Ptet ­HIV [11] with *Sph*I and *Nde*I (746 bp). The resulting constructs (9396 bp) are referred to as pKtCM-Ptet-n9-cm and pKtCM-Ptet-AT9-cm. Thus, genes encoding 9-CM under control of the P*_tet_* promoter [12] were introduced into the genome of the CM-deficient *E. coli* strain KA12 at the *kdgK* locus in a RecA-dependent manner according to published protocols [10]. Correct recombination with the chromosome was subsequently verified using PCR and restriction digestion analysis [10]. A scheme of the analysis strategy and the corresponding agarose gel, including the relevant primers and restriction enzymes, is shown in **Figure S8**. The recombinant strains were then transformed with a helper plasmid, pKIMP-UAUC [1], which sup­plies additional enzymes of the shikimate pathway, to yield KA12-n9-cm and KA12-AT9-cm.

#### Directed evolution in pulse-feed alternating turbidostats

Pulse-feed alternating turbidostats [13] were operated at Genoscope in Evry, France. For initial inoculation of the reactors, *E. coli* strain KA12-n9-cm harboring a chro­mosomal copy of *n9-cm* was grown in rich medium, washed with CM-medium (composition see **Table S4**), resuspended in CM-medium to an OD_600_ of 0.5, and transferred into the growth chamber of a turbidostat (19.5 mL volume). At regular intervals, a conditional pulse of fresh medium was delivered (if OD_600_ > 0.3) with concomitant draining of an equivalent volume, resulting in a 10% dilution of the culture with each pulse. To prevent enrichment of *E. coli* cells adhering to surfaces and thereby evading dilution [14], the growing culture was transferred to a backup chamber once every 24 hours while the growth chamber was sterilized with NaOH, rinsed, and emptied. At one point, it was necessary to re-initiate a turbidostat, which was accomplished with a 4 mL sample of the previous culture. Operation temperatures ranged from 25 °C to 42 °C according to **Figure 2** of the main text.

Selections with the AT-rich gene were conducted with plasmid pKECMT-AT9-cm, where *AT9-cm* is under control of the *trc* promoter, which is constitutive in the absence of the *lac* repressor protein [15]. KA12/pKIMP-UAUC cells were trans­formed with pKECMT-AT9-cm, and plated on rich medium in the presence of 150 µg/mL Na-ampicillin (amp) and 30 µg/mL chloramphenicol (cam). Transformants were subsequently adapted to reduced CM-medium (**Table S4**, no tetracycline added) supplemented with 20 µg/mL L-tyrosine and 20 µg/mL L-phenylalanine, and streaked on selective plates (CM-medium lacking Tyr and Phe). After growth for one week at 30 °C, single colonies were used to inoculate a liquid culture in selective reduced CM-medium (lacking Tyr and Phe), followed by incubation at 30 °C for 72 hours. With this culture, two separate pulse-feed alternating turbidostats were inoculated at an initial OD_600_ of 1.46. After 45 days of continuous growth under selective conditions (reduced CM-medium in the absence of Tyr, Phe and tetracycline), the *AT9-cm* gene of four and five clones from the individual cultures were se­quenced and analyzed by restriction digestion of their CM-encoding plasmids (**Figure S7**).

### *In vivo* complementation assays

CM complementation experiments with chromosomally and plasmid-encoded CMs were performed according to previously published procedures [1,8]. Briefly, *E. coli* cells harboring different CM constructs were plated on M9c­ agar plates [16] supplemented with 20 µg/mL cam, and variable amounts of tetracycline (0-2000 ng/mL) to induce the expression of CM genes under control of the *P_tet_* promoter. Non-selective control plates also included 20 µg/mL each of L-Tyr and L-Phe, and, in some cases, selective plates were supplemented with 20 µg/mL L-Phe as well. Growth from “3-dimensional” streak-outs was assessed using the arbitrary scale shown in **Table S5**. Clones selected from random cassette based libraries were subcloned into fresh pKT acceptor vector prior to performing *in vivo* complementation assays.

### Library construction and selection

Library selections on solid media were performed with CM genes under control of the P*_tet_* promoter in pKT vectors. Cassette mutagenesis at positions 30 and 61 was performed using over­lap extension PCR [17] of three fragments from pMG211-n9-cm. Individual reac­tions with primer pairs T7/30r, 30fx/61rx, 61f/T7TR were purified by gel elec­trophoresis, and assembled using the following protocol: Equimolar amounts of the fragments were first assembled in the absence of flanking primers for four cycles (95 °C for 30 sec, 50 °C for 1 min, 72 °C for 10 min). The PCR was completed after addition of T7 and T7TR primers for 30 cycles (95 °C for 30 sec, 50 °C for 1 min, 72 °C for 30 sec) and 5 min final extension at 72 °C. Products (508 bp) were isolated with a NucleoSpin PCR purification kit (Machery Nagel), digested with *Nde*I and *Xho*I, and ligated as a 282 bp fragment into the previously dephosphorylated (CIP, New England Biolabs) 3338 bp *Nde*I-*Xho*I-fragment of pKT over 24 hours at 16 °C using 8 U/µL T4 DNA ligase and a roughly 4:1 molar ratio of insert to vector on a 1 µg scale. Ligations were purified by phenol/chloroform extraction and concentrated using Vivacon 500 centrifugal filter devices (Sartorius, 30,000 MWCO). Approximately 500 ng of each library were transformed into electro­competent KA12/pKIMP-UAUC cells (0.1 cm cuvettes, 1.25 kV, 200 Ω, 25 µF). Cells were recovered in 16 mL SOC medium after 1 hour at 30 °C, washed three times with 1xM9 salts [18], and aliquots were plated on M9c agar [16] supplemented with various concentrations of tetracycline (0-2000 ng/mL). Fractions of the population (0.1 % and 0.01 %) were plated on M9c agar containing L-Phe and L-Tyr to evaluate library size and quality. Typical libraries contained 10^5^ clones. When ligations were performed in the absence of insert, less than 100 clones were found on non-selective control plates.

### Protein production and purification

For protein production, KA13 cells were freshly transformed with CM-encoding pMG211 plasmids, and incubated at 30 °C in 5 mL LB for 10 hours. Two mL of these dilute cultures were used to inoculate 500 mL LB medium containing 150 µg/mL amp and 1 % w/v D(+)glucose. The cells were grown to an OD_600_ of 0.6-0.8 at 30 °C and 230 rpm, followed by gene expression (induction with IPTG at a final concentration of 0.25 mM) for approximately 10 hours at 20 °C. Thereafter, cells were harvested by centrifugation, washed with 50 mL PBS (10 mM NaH_2_PO_4_, pH 6.5, 160 mM NaCl), and stored at -20 °C.

To purify the CM variants, cell pellets were thawed in 25 mL PBS contain­ing 20 mM imidazole and supplied with a spatula tip of lysozyme and DNase I. After 60 min on ice, the cells were lysed by sonication with 18 x 10 s bursts with 10 s pauses in between. Cell debris was centrifuged at 4000 g and 4 °C for 20 min, and the cleared lysates were loaded on Ni-NTA columns (5 mL slurry, Sigma). After washing with 10 column volumes of PBS containing 20 mM imidazole, His-tagged proteins were eluted with PBS containing 250 mM imidazole. Protein-containing fractions, identified by treating aliquots with Coomassie plus (Thermo Fisher), were pooled and purified further by size-exclusion chromatography on a Superdex 75 HiLoad 26-60 column running in PBS, monitored at 220 nm. The dimer peak was isolated and concentrated using centrifugal filter units (Millipore) with a 3 kDa cutoff. Protein samples were analyzed by SDS-PAGE and LC-MS (Finnigan LCQ Deca; 9-CM M+H^+^ calculated: 12670.0, observed: 12670 Da; 10-CM M+H^+^ calculated: 12657.9, observed: 12659 Da; 11-CM M+H^+^ calculated: 12643.9 observed: 12644 Da), and stored at 4 °C. Protein concentrations were determined with Coomassie plus (Thermo Fisher) using bovine serum albumin (BSA) as a standard.

### Protein characterization

Circular dichroism (CD) spectra of 16 µM CM in PBS were recorded at 25 °C on an Aviv 202 spectropolarimeter between 190 and 260 nm in 1 nm steps with an averaging time of 2 s in a 2 mm cuvette. Each sample was scanned three times, and the resulting spectra were averaged and corrected for background signals. Thermal denaturation of 16 µM CM was monitored from 10 to 95 °C at 222 nm. The rate of heating was 1 °C /min with 0.5 °C steps, and the equilibration and averaging times were 60 sec.

Chemical denaturation was performed with 12.5 µM protein us­ing guanidinium chloride (GdmCl) or urea at 25 °C in PBS, pH 6.5, and moni­tored by CD at 222 nm for 60 sec per sample. Protein solutions were added to mixtures of PBS and 8 M GdmCl or 10 M urea, and incubated in a water bath at room temperature for approximately 15 min. Concentrations of de­naturant stocks were verified using standard procedures [19,20]. The plateau regions at very low and very high denaturant concentrations were fit to a straight line to allow conversion of CD signals into the fraction unfolded (f_U_), which was sub­sequently converted into ∆G_U_^0^ using a dimeric model [5]. The ∆G_U_^0^ values for the transition region were fit linearly as a function of the concentration of denaturant to extrapolate to the free energy of unfolding in water, ∆G_U_^0^(H_2_O), and to determine the cooperativity of unfolding, *m*, from the slope. For visualiza­tion, the ∆G_U_^0^(H_2_O) and m values from the linear fit were used to calculate a theoretical f_U_ at each concentration of denaturant, plotted as a solid line in **Figure 4A** [5].

### Kinetic assays

CM activity measurements were conducted according to previously published procedures [5], with the following alterations: All measurements were performed in the presence of 0.1 mg/mL BSA at 0.4 or 1 µM enzyme concentration in PBS at pH 6.5 and 30 °C (full kinetics) or in 20 mM acetate containing 100 mM NaCl at pH 5 (only at low [S]). Chorismate [21] was HPLC-puriﬁed on a Nucleosil 100-7 C-18 reverse phase column, using a gradient from 5 to 40% MeCN (containing 0.05% TFA) in H_2_O (containing 0.1 % TFA), lyophilized, and dissolved in H_2_O and adjusted to neutral pH with 2 M NaOH. Chorismate concentrations were determined from the difference in absorption at 274 nm of an aliquot upon complete conversion to prephenate with wild-type MjCM, produced according to previously published procedures [5].

**Limited Proteolysis**

50 μM CM samples were digested with 0.01 mg/ml trypsin (9000 U/mg, Sigma) in PBS pH 6.5 at 25°C. The proteolysis reactions were quenched at defined intervals by the addition of 2 µl of SDS-PAGE loading buffer (250 mM Tris, pH 6.8, 500 mM DTT, 10% (w/v) SDS, 0.5% (w/v) Bromophenol Blue, 50% (w/v) glycerol) into 8 µl of the proteolysis reaction. The digested samples were separated by SDS-PAGE on a High Density PhastGel (GE Healthcare). For LC-MS analysis, 50 μM 11-CM were incubated with 0.01 mg/ml trypsin for 1 h. Then, the reaction was quenched with 2 mM phenylmethanesulfonylfluoride and injected into an Ultimate 3000 HPLC (Dionex) equipped with an Atlantis T3 3 μm particle size 2.1 x 50 mm reverse-phase column (Waters) and eluted with a linear gradient from 95% solvent A: 5% solvent B to 35% solvent A: 65% solvent B for 9 min (solvent A, 0.1% TFA aqueous solution; solvent B, MeCN). The masses of 11-CM fragments were determined on a Finnigan LCQ Deca mass spectrometer (Thermo) connected to the HPLC.

### Western Blotting

CM variants were produced from pMG211 plasmids in KA12 in the presence of 1000 ng/mL tetracycline. Cells were cooled to 4°C in their exponential growth phase and harvested by centrifugation. Upon resuspension in PBS (normalization to a final OD_600_ = 3), cells were lysed by sonication, and cell debris removed by centrifugation. Crude protein supernatants were separated on a 20% SDS-polyacrylamide gel, and subsequently blotted onto a nitrocellulose membrane using the PhastSystem (GE Healthcare). The membrane was incubated with anti-histidine tag antibody conjugated with horseradish peroxidase (Penta-His HRP conjugate, Qiagen). Chemiluminescence was generated with the Immobilon Western Chemiluminescence HRP Substrate (Millipore), and detected by exposure to Amersham Hyperfilm ECL (GE Healthcare).

### Molecular dynamics simulations

All simulations and energy minimizations were carried out using the GROMOS05 biomolecular simulation software [22] and the GROMOS 53A6 force field [23,24]. Initial coordinates of EcCM were taken from the X-ray structure (PDB code 1ECM) [25], which includes a transition state analog inhibitor (TSA). The 9‑CM sequence was homology modeled onto the EcCM structure using the I-TASSER web server [26]. The best dimeric model was chosen (model 2) and the His_6_ tag removed. The coordinates of each subunit were aligned to those of the corresponding subunit of EcCM using the VMD MultiSeq tool [27,28]. Hydrogens were added using the GROMOS++ gch program [29], with the protonation state of ionizable residues chosen according to a pH of 6.5. Additional residues were added to the N-terminus of subunit A (Ser-Glu) and subunit B (Ser-Glu-Asn) using Pymol [30]. Coordinates for 10‑CM and 11‑CM were created from those of 9-CM using the VMD Mutator tool [31]. For the simplified mutases, the TSA molecules were added to each subunit using the coordinates from the EcCM structure. The parameters for the TSA were assigned by analogy with the building blocks of standard molecules with similar chemical structure in the GROMOS 53A6 force field. Details of the choices of force field parameters are available upon request.

Each protein/ligand system was subjected to 2000 steps of steepest descent energy minimization, then solvated in a rectangular box with a minimum distance of 1.2 nm from any solute atom to the edge of the box. The simple point charge (SPC) [32] water model was used, and periodic boundary conditions were applied. All simulations were initiated with the following equilibration scheme: first, the initial velocities were randomly generated from a Maxwell-Boltzmann distribution at 60 K. All solute atoms were restrained to their positions in the corresponding energy-minimized structure through a harmonic potential energy term with a force constant of 2.5 × 10^4^ kJ mol^-1^ nm^-2^. Each system was simulated with these settings for 20 ps, followed by three consecutive 20 ps simulations; prior to each run, the temperature was raised by 60 K, and the force constant for the positional restraints was reduced by a factor of 10. The position restraints were then removed, and a further 20 ps simulation carried out, raising the temperature to 293 K. The final structure was used as the starting configuration for a 10 ns production run of each system at 293 K. The SHAKE algorithm [33] was used with a geometric precision of 10^4^  to constrain bond lengths, allowing for an integration time step of 2 fs. The center of mass motion was removed every 1000 time steps. The temperature and atmospheric pressure were kept constant using a weak coupling approach [34] with relaxation times τ_T_ = 0.1 ps and τ_p_ = 0.5 ps and an isothermal compressibility of 4.575 × 10^4^ (kJ mol^-1^ nm^3^)^-1^. Non-covalent interactions were calculated using a triple-range cutoff scheme. The interactions within a cutoff distance of 0.8 nm were calculated at every step from a pair list that was updated every fifth time step. At this point, interactions between atoms (of charge groups) within 1.4 nm were also calculated and were kept constant between updates. To account for the influence of the dielectric medium outside the cutoff sphere of 1.4 nm, a reaction field force based on a relative dielectric permittivity ε of 61 [35] was added.

Analysis of the energy-minimized structures and MD trajectories was carried out using the GROMOS++ suite of programs [29]. The secondary structure content was calculated according to the rules of Kabsch and Sander [36].

### References

1. Kast P, Asif-Ullah M, Jiang N, Hilvert D (1996) Exploring the active site of chorismate mutase by combinatorial mutagenesis and selection: The importance of electrostatic catalysis. Proc Natl Acad Sci USA 93: 5043-5048.

2. Kast P, Asif-Ullah M, Hilvert D (1996) Is chorismate mutase a prototypic entropy trap? - activation parameters for the *Bacillus subtilis* enzyme. Tetrahedron Lett 37: 2691-2694.

3. MacBeath G, Kast P (1998) UGA read-through artifacts - when popular gene expression systems need a pATCH. BioTechniques 24: 789-794.

4. Sambrook J, Fritsch EF, Maniatis T (1989) Molecular cloning: A laboratory manual. Cold Spring Harbor, NY: Cold Spring Harbor Laboratory Press.

5. MacBeath G, Kast P, Hilvert D (1998) A small, thermostable, and monofunctional chorismate mutase from the archaeon *Methanococcus jannaschii*. Biochemistry 37: 10062-10073.

6. Walter KU, Vamvaca K, Hilvert D (2005) An active enzyme constructed from a 9-amino acid alphabet. J Biol Chem 280: 37742-37746.

7. Sasso S, Ramakrishnan C, Gamper M, Hilvert D, Kast P (2005) Characterization of the secreted chorismate mutase from the pathogen *Mycobacterium tuberculosis*. FEBS J 272: 375-389.

8. Neuenschwander M, Butz M, Heintz C, Kast P, Hilvert D (2007) A simple selection strategy for evolving highly efficient enzymes. Nat Biotechnol 25: 1145-1147.

9. MacBeath G, Kast P, Hilvert D (1998) Exploring sequence constraints on an interhelical turn using *in vivo* selection for catalytic activity. Protein Sci 7: 325-335.

10. Gamper M, Kast P (2005) Strategy for chromosomal gene targeting in RecA-deficient *Escherichia coli* strains. BioTechniques 38: 405-408.

11. Neuenschwander M (2007) High-throughput assays for detecting enzymatic activities in living cells. Zürich: ETH Zürich.

12. Lutz R, Bujard H (1997) Independent and tight regulation of transcriptional units in *Escherichia coli* via the LacR/O, the TetR/O and AraC/I_1_-I_2_ regulatory elements. Nucl Acids Res 25: 1203-1210.

13. Mutzel R, Marlière P (2000) Method and device for selecting accelerated proliferation of living cells in suspension. WO2000034433.

14. de Crécy-Lagard V, Bellalou J, Mutzel R, Marlière P (2001) Long term adaptation of a microbial population to a permanent metabolic constraint: Overcoming thymineless death by experimental evolution of *Escherichia coli*. BMC Biotechnol 1: 10.

15. de Boer HA, Comstock LJ, Vasser M (1983) The *tac* promoter: A functional hybrid derived from the *trp* and *lac* promoters. Proc Natl Acad Sci USA 80: 21-25.

16. Gamper M, Hilvert D, Kast P (2000) Probing the role of the C-terminus of *Bacillus subtilis* chorismate mutase by a novel random protein-termination strategy. Biochemistry 39: 14087-14094.

17. Higuchi R, Krummel B, Saiki R (1988) A general method of *in vitro* preparation and specific mutagenesis of DNA fragments: Study of protein and DNA interactions. Nucleic Acids Res 16: 7351-7367.

18. Kleeb AC, Edalat MH, Gamper M, Haugstetter J, Giger L, et al. (2007) Metabolic engineering of a genetic selection system with tunable stringency. Proc Natl Acad Sci USA 104: 13907-13912.

19. Warren JR, Gordon JA (1966) On the refractive indices of aqueous solutions of urea. J Phys Chem 70: 297-300.

20. Nozaki Y (1972) The preparation of guanidine hydrochloride. Methods Enzymol 26: 43-50.

21. Grisostomi C, Kast P, Pulido R, Huynh J, Hilvert D (1997) Efficient *in vivo* synthesis and rapid purification of chorismic acid using an engineered *Escherichia coli* strain. Bioorg Chem 25: 297-305.

22. Christen M, Hünenberger PH, Bakowies D, Baron R, Bürgi R, et al. (2005) The GROMOS software for biomolecular simulation: GROMOS05. J Comput Chem 26: 1719-1751.

23. Oostenbrink C, Villa A, Mark AE, van Gunsteren WF (2004) A biomolecular force field based on the free enthalpy of hydration and solvation: The GROMOS force-field parameter sets 53A5 and 53A6. J Comput Chem 25: 1656-1676.

24. Oostenbrink C, Soares TA, van der Vegt NFA, van Gunsteren WF (2005) Validation of the 53A6 GROMOS force field Eur Biophys J 34: 273-284.

25. Lee AY, Karplus PA, Ganem B, Clardy J (1995) Atomic structure of the buried catalytic pocket of *Escherichia coli* chorismate mutase. J Am Chem Soc 117: 3627-3628.

26. Zhang Y (2008) I-TASSER server for protein 3D structure prediction. BMC Bioinf 9: 40.

27. Humphrey W, Dalke A, Schulten K (1996) VMD - visual molecular dynamics. J Molec Graphics 14: 33-38.

28. Eargle J, Wright D, Luthey-Schulten Z (2006) Multiple alignment of protein structures and sequences for VMD. Bioinformatics 22: 504-506.

29. Eichenberger AP, Allison JR, Dolenc J, Geerke DP, Horta BAC, et al. (2011) GROMOS++ software for the analysis of biomolecular simulation trajectories. J Chem Theory Comput 7: 3379-3390.

30. Schrödinger L (2010) The pymol molecular graphics system, version 1.3r1.

31. Humphrey W, Dalke A, Schulten K (1996) VMD - visual molecular dynamics. J Molec Graphics 14: 33-38.

32. Berendsen HJC, Postma JPM, van Gunsteren WF, Hermans J (1981) Interaction models for water in relation to protein hydration. In: Pullmann B, editor. Intermolecular forces. Dordrecht, The Netherlands: Reidel. pp. 331-342.

33. Ryckaert J-P, Ciccotti G, Berendsen HJC (1977) Numerical integration of the cartesian equations of motion of a system with constraints: Molecular dynamics of n-Alkanes. J Comp Phys 23: 327-341.

34. Berendsen HJC, Postma JPM, van Gunsteren WF, DiNola A, Haak JR (1984) Molecular dynamics with coupling to an external bath. J Chem Phys 81: 3684-3690.

35. Heinz TN, van Gunsteren WF, Hünenberger PH (2001) Comparison of four methods to compute the dielectric permittivity of liquids from molecular dynamics simulations. J Chem Phys 115: 1125-1136.

36. Kabsch W, Sanders C (1983) Dictionary of protein secondary structure: Pattern recognition of hydrogen-bonded and geometrical features. Biopolymers 22: 2577-2637.
